# Great Cause—Small Effect: Undeclared Genetically Engineered Orange Petunias Harbor an Inefficient Dihydroflavonol 4-Reductase

**DOI:** 10.3389/fpls.2018.00149

**Published:** 2018-02-28

**Authors:** Christian Haselmair-Gosch, Silvija Miosic, Daria Nitarska, Barbara L. Roth, Benjamin Walliser, Renate Paltram, Rares C. Lucaciu, Lukas Eidenberger, Thomas Rattei, Klaus Olbricht, Karl Stich, Heidi Halbwirth

**Affiliations:** ^1^Institute of Chemical, Environmental and Bioscience Engineering, Technische Universität Wien, Vienna, Austria; ^2^Department of Microbiology and Ecosystem Science, University of Vienna, Vienna, Austria; ^3^Thaer-Institute of Agricultural and Horticultural Sciences Humboldt University Berlin, Berlin, Germany

**Keywords:** *Petunia* × *hybrida*, *Zea mays*, dihydroflavonol 4-reductase, *A*_*1*_ type 2 allele, anthocyanin, pelargonidin, orange flower color, transgenic plant

## Abstract

A recall campaign for commercial, orange flowering petunia varieties in spring 2017 caused economic losses worldwide. The orange varieties were identified as undeclared genetically engineered (GE)-plants, harboring a maize dihydroflavonol 4-reductase (*DFR, A*_1_), which was used in former scientific transgenic breeding attempts to enable formation of orange pelargonidin derivatives from the precursor dihydrokaempferol (DHK) in petunia. How and when the *A*_*1*_ cDNA entered the commercial breeding process is unclear. We provide an in-depth analysis of three orange petunia varieties, released by breeders from three countries, with respect to their transgenic construct, transcriptomes, anthocyanin composition, and flavonoid metabolism at the level of selected enzymes and genes. The two possible sources of the *A*_*1*_ cDNA in the undeclared GE-petunia can be discriminated by PCR. A special version of the *A*_*1*_ gene, the *A*_*1*_ type 2 allele, is present, which includes, at the 3′-end, an additional 144 bp segment from the non-viral transposable *Cin4-1* sequence, which does not add any functional advantage with respect to DFR activity. This unequivocally points at the first scientific GE-petunia from the 1980s as the *A*_*1*_ source, which is further underpinned e.g., by the presence of specific restriction sites, parts of the untranslated sequences, and the same arrangement of the building blocks of the transformation plasmid used. Surprisingly, however, the GE-petunia cannot be distinguished from native red and blue varieties by their ability to convert DHK in common *in vitro* enzyme assays, as DHK is an inadequate substrate for both the petunia and maize DFR. Recombinant maize DFR underpins the low DHK acceptance, and, thus, the strikingly limited suitability of the *A*_*1*_ protein for a transgenic approach for breeding pelargonidin-based flower color. The effect of single amino acid mutations on the substrate specificity of DFRs is demonstrated. Expression of the *A*_*1*_ gene is generally lower than the petunia *DFR* expression despite being under the control of the strong, constitutive p*35S* promoter. We show that a rare constellation in flavonoid metabolism—absence or strongly reduced activity of both flavonol synthase and B-ring hydroxylating enzymes—allows pelargonidin formation in the presence of DFRs with poor DHK acceptance.

## Introduction

**Figure 1:**
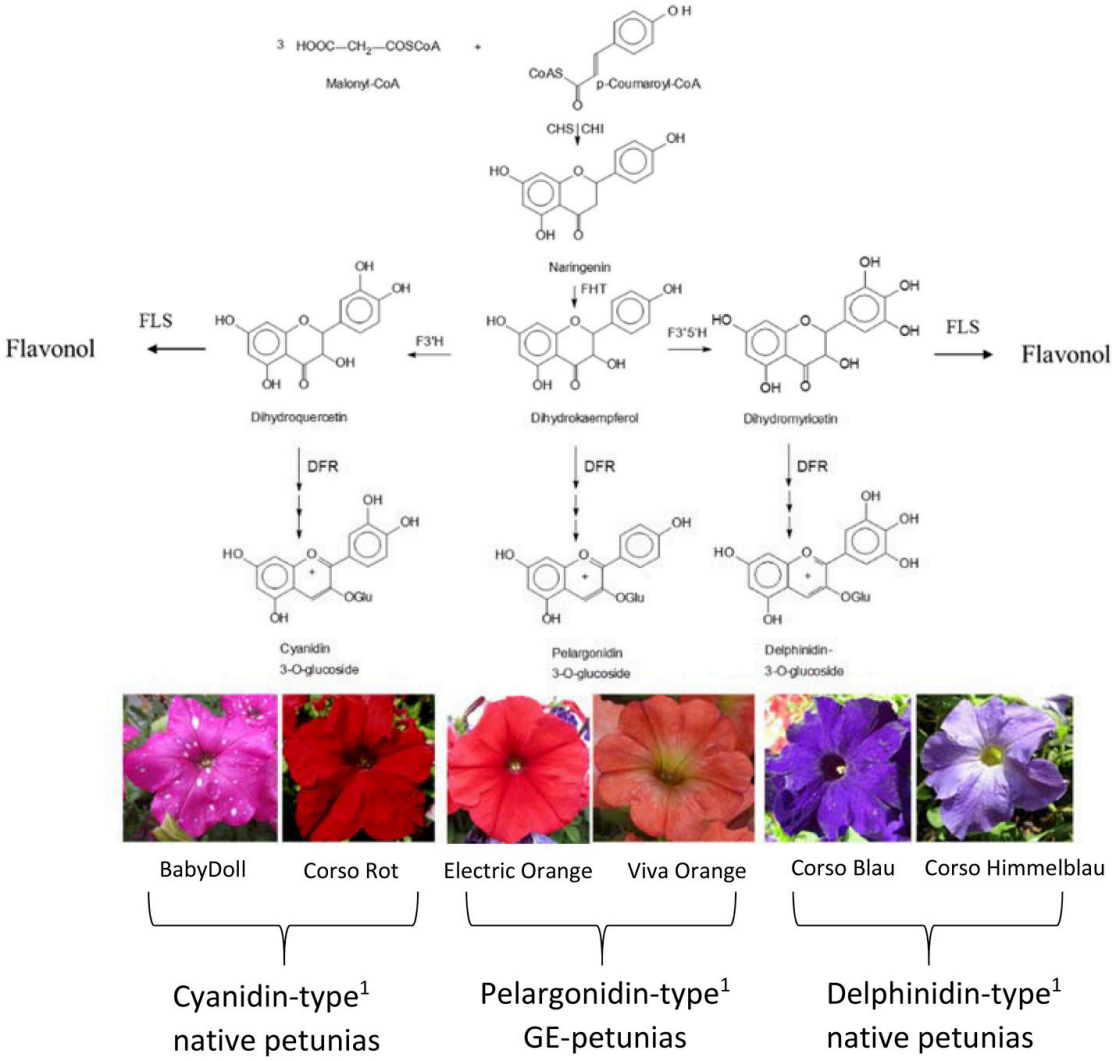
Simplified flavonoid pathway demonstrating the influence of the B-ring hydroxylation pattern on the establishment of petunia flower color. ^1^Petals contain prevalently the respective anthocyanidin type. CHS, Chalcone synthase; CHI, chalcone isomerase; DFR, dihydroflavonol 4-reductase; FHT, flavanone 3-hydroxylase; F3′H, flavonoid 3′-hydroxylase; F3′5′H, flavonoid 3′,5′-hydroxylase; FLS, flavonol synthase.

The color of anthocyanin pigments is determined by their B-ring hydroxylation pattern (Figure 1), ranging from orange to bright red (one hydroxy group), dark red to magenta (two hydroxy groups), and violet to blue (three hydroxy groups; Halbwirth, 2010). This basically depends on two factors, which have both been exploited by biotechnological methods to influence flower color (Meyer et al., 1986; Tanaka et al., 2009, 2010): the presence of enzymes introducing hydroxy groups vicinal to that in position 4′ [flavonoid 3′-hydroxylase (F3′H) and flavonoid 3′5′-hydroxylase (F3′5′H)], and the substrate specificity of DFR (Winkel-Shirley, 2001).

Important ornamental plants such as petunia, African violet and cyclamen do not naturally produce orange/bright-red flowers (Johnson et al., 1999) because they lack the ability to synthesize pelargonidin-type anthocyanin pigments. This is based on the presence of a substrate specific dihydroflavonol 4-reductase (DFR) enzyme, which does not accept the essential precursor, dihydrokaempferol (DHK), as a substrate.

DFR is an oxidoreductase (EC 1.1.1.219) that catalyzes the NADPH dependent stereospecific reduction of the keto group of (+)-(*2R,3R*)-dihydroflavonols in position 4 to the respective (*2R,3S,4S*)-flavan-2,3-*trans*-3,4-*cis*-diols (leucoanthocyanidins), as well as the reverse reaction in the presence of NADP^+^ (Halbwirth et al., 2006; Petit et al., 2007). DFR is the first of the so-called “late” enzymes of the flavonoid pathway which shows a major impact on the formation of anthocyanin pigments, flavan 3-ols and flavonols. DFR provides the immediate precursors for the formation of anthocyanidins and flavan 3-ols, the building blocks of condensed tannins. On the formation of flavonols, DFR has an indirect effect. DFR competes with flavonol synthase (FLS), which opens a side branch of the anthocyanin pathway, for common substrates (Winkel-Shirley, 2001; Figure 1). Several DFRs can convert dihydroflavonols irrespective of their hydroxylation pattern, but petunia possesses a DFR that does not convert DHK into leucopelargonidin. In the 1980s and 1990s genetically engineered (GE)-petunias with orange flowers were created by introducing either a maize DFR encoded by the *A*_*1*_ gene (Meyer et al., 1986; Elomaa et al., 1995) or a gerbera DFR (Elomaa et al., 1995).

The petunia belongs to the predominant balcony and bedding plants worldwide. A few years ago petunia varieties, showing a novel orange flower color, started to appear on the market and were swiftly adopted in private and public flower arrangements, in Europe and the US. Recently, the vast majority of them turned out to be genetically modified, after PCR-screening for the *35S*-promoter and the *A*_*1*_ gene (Bashandy and Teeri, 2017; David, 2017; Servick, 2017).

We selected three varieties, released by breeders from three countries, for an-in-depth investigation of the presence and nature of a transgenic construct and its impact on the flavonoid metabolism. We show that they all carry the same construct, and that this can be traced back to the first GE-petunia (Meyer et al., 1986) with near absolute certainty. But surprisingly, the orange petunias were not characterized by a drastically changed DFR substrate specificity compared to common red and blue petunia flowers, as would have been expected. We aimed on elucidating this paradox and demonstrate that the orange petunia owe their color primarily to a rare biochemical background. We underpin this by flavonoid analyses together with enzyme assays and expression and transcriptome studies.

## Materials and methods

### Material

**Figure 2:**
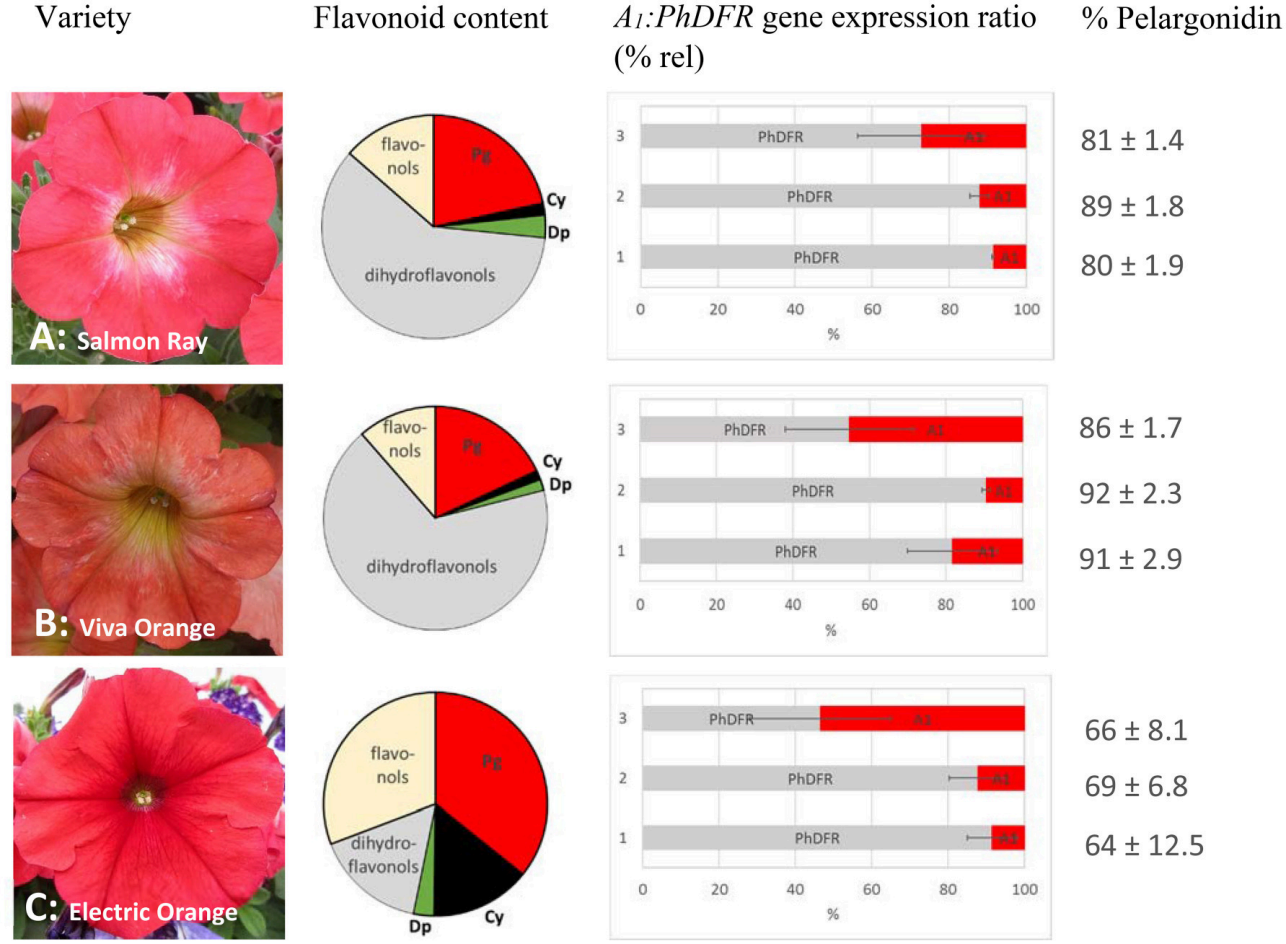
Flavonoid content, *A*_*1*_*:PhDFR* gene expression ratio and relative pelargonidin content of flowers from *Petunia* × *hybrida* cv. Salmon Ray **(A)**, cv. Viva Orange **(B)**, and cv. Electric Orange **(C)**. Pie chart (bright red: pelargonidin (Pg), black: cyanidin (Cy), green: delphinidin (Dp), gray: dihydroflavonols, yellow: flavonols) of flavonoid contents (for absolute values see Table 1). Bar chart with the ratio of *A1:PhDFR* gene expression in % rel for each developmental stage (1: buds of 0.6–3 cm length, 2: buds of 3–5 cm length, 3: open flowers). Standard deviation (error bars) in *A*_*1*_ and *PhDFR* gene expression ratios for each developmental stage are shown. Portions of pelargonidin based pigments of the anthocyanidins in total are given in % pelargonidin in the three stages of each cultivar.

Flowers (stage 1: buds of 0.6–3 cm length, stage 2: buds of 3–5 cm length, stage 3: open flowers) of cv. Salmon Ray (Danziger, Moshav Mishmar Hashiva, Israel), cv. Viva Orange (Florensis, Ambacht, The Netherlands), and cv. Electric Orange (Selecta One, Stuttgart, Germany) were harvested in the summers 2015–2017. Non-transgenic control plants of *Petunia* × *hybrida* cv. BabyDoll were obtained from Selecta One, cvs. Corso Rot, Corso Blau and Blackberry were purchased from Austrosaat (Vienna, Austria). The plant material was harvested from balcony pots or garden beddings, shock-frozen and kept at −80°C until analysis. Images of the petunia varieties are found in Figures 1, 2 and Supplementary Figure S3.

Reference compounds (cyanidin, delphinidin, malvidin, pelargonidin, peonidin, petunidin, dihydromyricetin, dihydroquercetin, kaempferol, myricetin, and quercetin) were purchased from Extrasynthese (Genay, France), dihydrokaempferol from Sigma Aldrich (Vienna, Austria). Radiolabeled substrates were synthesized as previously described (Halbwirth et al., 2006).

**Table 1:** Flavonoid composition ([μg/g FW] and % rel) of methanolic extracts of three orange flowering petunias after acidic and enzymatic hydrolysis in comparison to the non-GE cultivars Corso Rot (red), Corso Blau (blue) and BabyDoll (pink with white dots).

Pigment composition after acidic or enzymatic hydrolysis^*^		**Salmon ray**	**Viva Orange**	**Electric Orange**	**Corso Rot**	**Corso Blau**	**BabyDoll**
Total anthocyanidins^**^	[μg/g FW]	684.6 ± 299.6	522.7 ± 2.1	684.6 ± 299.6	552.0 ± 44.7	737.5 ± 194.4	959.3 ± 148.4
Pelargonidin type	[μg/g FW]	555.5 ± 193.4	448.5 ± 93.0	474.6 ± 314.2	n.d.	n.d.	n.d.
Cyanidin type	[μg/g FW]	43.0 ± 32.0	32.7 ± 7.3	191.7 ± 74.2	521.8 ± 45.0	n.d.	853.7 ± 123.8
Delphinidin type	[μg/g FW]	86.0 ± 15.7	41.5 ± 3.8	36.9 ± 43.9	30.3 ± 14.4	737.5 ± 194.4	105.6 ± 24.6
Total dihydroflavonols	[μg/g FW]	1, 538.9 ± 1, 040.6	1, 680.2 ± 88.8	212.4 ± 47.8	455.0 ± 66.9	n.d.	188.2 ± 32.3
Dihydrokaempferol (DHK)	[μg/g FW]	1, 502.4 ± 1, 040.61	1, 652.5 ± 88.1	179.9 ± 34.0	n.d.	n.d.	n.d.
Dihydroquercetin (DHQ)	[μg/g FW]	36.5 ± 29.5	27.8 ± 10.6	32.5 ± 26.2	455.0 ± 66.9	n.d.	188.2 ± 32.3
Dihydromyricetin (DHM)	[μg/g FW]	n.d.	n.d.	n.d.	n.d.	n.d.	n.d.
Total flavonols	[μg/g FW]	348.8 ± 276.2	284.1 ± 130.5	408.53 ± 190.6	857.2 ± 189.6	1, 458.3 ± 42.4	1, 942.1 ± 232.4
Kaempferol	[μg/g FW]	298.3 ± 205.3	284.1 ± 130.5	180.3 ± 27.7	n.d.	n.d.	168.9 ± 30.5
Quercetin	[μg/g FW]	50.5 ± 71.4	n.d.	228.1 ± 164.2	857.2 ± 189.6	1, 407.1 ± 38.5	1, 773.0 ± 199.9
Myricetin	[μg/g FW]	n.d.	n.d.	n.d.	n.d.	51.2.±3.9	n.d.
Dihydroflavonols	%	57 ± 4.1	68 ± 4.1	17±.1.5	29±.6.5	n.d.	6±.1.3
Flavonols	%	12 ± 2.7	11 ± 4.9	41 ± 2.1	33 ± 4.8	66 ± 5.1	63±.5.1
Anthocyanidins^**^	%	31 ± 6.4	21 ± 1.2	42 ± 3.7	32 ± 7.9	34 ± 5.1	31±.6.4
Pelargonidin type	%	81 ± 1.4	86 ± 1.7	66 ± 8.1	n.d.	n.d.	n.d.
Cyanidin type	%	6±.2.2	6±.1.2	30±.8.3	95±.2.7	n.d.	11±.2.9
Delphinidin type	%	13±.2.2	8±.0.8	4±.3.1	5±.2.7	100±.2.7	89±.1.7

*Pictures of the cultivars are incorporated in Figure 1*.

* *Average values and standard deviations were calculated from at least three biological replications collected at different sites. Large standard deviations of absolute values [μg/g fresh weight (FW)] partially result from the strong variation of flavonoid contents with external factors such as lighting conditions. But even from the same plants, flowers with divergent color intensity could be collected (details not shown). Despite this, the relative distribution [%] between the flavonoid classes (anthocyanins, dihydroflavonols, and flavonols) and within anthocyanidins (pelargonidin-, cyanidin-, delphinidin based pigments) was quite stable*.

** *Methylated anthocyanidins are not shown separately, but were included according to their number of hydroxy groups in the delphinidin type (petunidin, malvidin) or cyandin type (peonidin) anthocyanidins*.

*n.d. not detected*.

### HPLC analysis

For analyzing the flavonoid class/anthocyanidin type composition in the petals, sugar moieties were removed by acidic or enzymatic hydrolysis. 1 g plant material was extracted with 1 ml 2 M hydrochloric acid in methanol. For anthocyanin analysis, 40 μl of the supernatant after centrifugation were incubated with 160 μl 4 N HCl for 60 min at 95°C. For analysis of other flavonoids, 20 μl of the supernatant were subjected to enzymatic hydrolysis by 10 U Naringinase (Sigma-Aldrich, Vienna, Austria) and hydrolysed for 20 min at 40°C in 0.1 M McIlvaine buffer pH 4. After hydrolysis, solid compounds were removed by centrifugation, and 4 μl of the supernatants were injected after filtration by 0.2 μm syringe filters.

HPLC analysis was performed on a Thermo Scientific Dionex UltiMate® 3000 RSLC System with DAD-3000RS Photodiode Array Detector (Thermo Scientific, Germany) using an Acclaim™ column RSLC 120 C18, 2.2 μm, 120Å, 2.1 × 150 mm (Dionex Bonded Silica Products: No. 071399) operated at 25°C. For analysis of anthocyanidins, elution solvents were (A) 10% formic acid and (B) 10% formic acid/22.5% acetonitrile/22.5% methanol in water (v/v) using a slightly modified method from Thermo Scientific Application note 281 (gradient: −10 to 0 min 9% B, 0–30 min 9–90% B, 30–40 min 90% B; flow rate 0.2 ml/min). For analysis of other flavonoids, elution solvents were (A) 0.1% formic acid and (B) 0.1% formic acid in acetonitrile (gradient: −3 to 0 min 20% B, 0–15 min 20–53% B, 15–20 min 53–95% B; 20–30 min 95% B, 31–35 min 20% B; flow rate 0.2 ml/min). Anthocyanidins were detected at 520 nm, other flavonoids at 290 nm. All compounds were identified by retention times and comparison of their UV-VIS spectra from 190 to 800 nm. The concentrations were calculated from the peak areas of samples and standard lines obtained with the respective reference compounds. Methylated anthocyanidins are not listed separately (Table 1), but were included according to their number of hydroxy groups in the delphinidin type (petunidin, malvidin) or cyandin type (peonidin) anthocyanidins. Relative contents of anthocyanidin types (% Pg/Cy/Dp based pigments was calculated from the [μg/g] values in Table 1. Flavonoid class distribution (% anthocyandins/flavonols/dihydroflavonols) were calculated from the [μg/g] values in Table 1 in relation to a mathematical total amount of flavonoids resulting from the three types.

### PCR, qPCR

Genomic DNA was obtained according to Lipp et al. (1999). mRNA was extracted with the μMACS mRNA isolation Kit (Miltenyi Biotec, Germany) and cDNA was synthesized as described (Thill et al., 2012). PCR and qPCR primers are listed in Supplementary Table S1. PCR was performed with the GoTaq DNA polymerase (Promega, Germany). Quantitative gene expression (at least in biological triplicates with three technical replications each) of *DFR, A*_1_*, F3*′*H*, and *FLS* in comparison to the *actin* reference gene (Mallona et al., 2010) were analyzed with a StepOnePlus system (Applied Biosystems, CA, USA) and the Luna® Universal qPCR Master Mix (New England Biolabs, Ipswich, UK). The relative expression ratio was calculated according to Pfaffl (2001). The efficiency of the PCR-reaction was determined on the basis of standard curves which were obtained by applying different DNA concentrations and calculated from the given slopes in the StepOne software according to equation *E* = 10^(−1/slope)^(Pfaffl, 2001). All qPCR primers had an efficiency between 90 and 110% (for amplification efficiencies see Supplementary Table S1). The product specificity was confirmed by melting curve analysis and gel electrophoresis. Sequencing of PCR products was done by a commercial supplier (Microsynth, Vienna, Austria).

### Transcriptome analysis

Plant material was harvested in summer 2016 and shock-frozen with liquid nitrogen. Preparation of rRNA, depleted RNA, random-primed cDNA and Illumina PE sequencing (50 million 150 bp, paired-end reads) was performed by a commercial supplier (vertis AG, Freising, Germany) on an Illumina NextSeq 500 system using 2 × 150 bp read length.

The random tagged prime RNA-seq data was first analyzed with the common NGS (next generation sequencing) analysis tools: First, the entire rRNA database provided by the tool was analyzed with sortmerna (v 2.1; Kopylova et al., 2012). From the remaining reads, the low-quality reads (below 20 quality score) were trimmed using trimmomatic (v 0.36) (Bolger et al., 2014) and the parameters TRAILING:20 AVGQUAL:20 SLIDINGWINDOW:5:20 MINLEN:75. Reads were mapped against the available *Petunia axillaris* genome (Bombarely et al., 2016), in which we incorporated the sequence of *A*_*1*_ (NCBI CAA28734) to allow a quantification of the transgene expression. FPKM (Fragments Per Kilobase of transcript per Million mapped reads) were obtained using the method for quantification of RNA expression RSEM (v 1.3.0) (Li and Dewey, 2011).

### Recombinant DFR

The DFR cDNA clone of *P. hybrida* was synthesized by GeneCust Europe (Dudelange, Luxembourg) based on the sequence available in the database (NCBI X15537). The *A*_*1*_ cDNA clone (NCBI CAA28734) from maize was provided by Udo Wienand (University of Hamburg, Germany). The cDNA clones were used for subcloning into the bacterial expression vector pGEX-6P-1 (GE Healthcare, Munich, Germany) for overexpression of the *DFRs* as GST-fusion proteins, as described previously (Gosch et al., 2014), using primers A_1_DFR-FL, A_1_DFR-FS, A_1_DFR-RL, A_1_DFR-RS, and PhDFR-FL, PhDFR-FS, PhDFR-RL, PhDFR-RS, respectively (Supplementary Table S2).

### Enzyme assays

DFR assays with recombinant enzyme or preparations from flowers were performed as described previously (Gosch et al., 2014).

### Site directed mutagenesis

Mutants were generated by use of the Q5® Site-Directed Mutagenesis Kit (NewEngland Biolabs, Vienna, Austria). Primers were designed using the NEBase Changer™ provided at http://nebasechanger.neb.com. The sequences are given in Supplementary Table S2. The integrity of the constructs was confirmed by commercial sequencing (Microsynth, Vienna, Austria).

### Statistical analysis

The statistical analysis of the qPCR data was performed using RStudio v 1.0.136 and R v 3.3.3 with the package “agricolae” (Ihaka and Gentleman, 1996; De Mendiburu, 2017). Shapiro-Wilk test was used for testing on normality (Razali and Wah, 2011). A Wilcoxon rank sum test was used for not normal distributed data and a paired *t*-test was used for normal distributed data, respectively. The correlation between pelargonidin content and the expression ratio of *PhDFR* was calculated using the Pearson correlation coefficient (Duncan, 1955). Group-wise comparison of gene expression between different developmental stages and varieties was calculated utilizing Duncan's new multiple range test (MRT) (Duncan, 1955). For all statistical significance tests, the significance level was set to 0.05 (5%).

## Results

### Pigment composition

We analyzed the pigment composition of three commercially available orange varieties. In all three, pelargonidin based pigments were the prevalent anthocyanins (pie chart in Figure 2, Table 1). Whereas cv. Salmon Ray and cv. Viva Orange contained more than 80% pelargonidin based pigments and only small amounts of cyanidin and delphinidin derivatives, cv. Electric Orange showed a relatively high content of cyanidin based pigments (30%), traces of delphinidin based pigments and 66% pelargonidin based pigments. The anthocyanin pattern was relatively stable during flower development and varied only to a minor extent between buds of different size and fully developed flowers (Figure 2 right). Common red, pink, or blue petunia varieties, which were analyzed as controls, accumulated cyanidin or delphinidin based pigments, depending on the color, but no pelargonidin based pigments could be detected (Table 1).

Besides anthocyanins, dihydroflavonols and flavonols were present in the petals (Figure 2, Table 1). In the orange cultivars Salmon Ray and Viva Orange, dihydroflavonols were the prevalent flavonoid class, with more than 55% of the total flavonoids, whereas relatively small amounts of flavonols (below 15%) could be found (Table 1, pie charts in Figure 2). As with the anthocyanins, cv. Electric Orange showed a somewhat different composition, with 41% flavonols and 17% dihydroflavonols. In the red cv. Corso Rot, concentrations of anthocyanins, dihydroflavonols and flavonols were almost equal, whereas the blue and pink cultivars contained more than 60% flavonols and no (cv. Corso Blau) or only traces of (cv. BabyDoll) dihydroflavonols (Table 1).

### Evidence for a genetic modification event and identification of the *A*_*1*_ source

**Figure 3:**
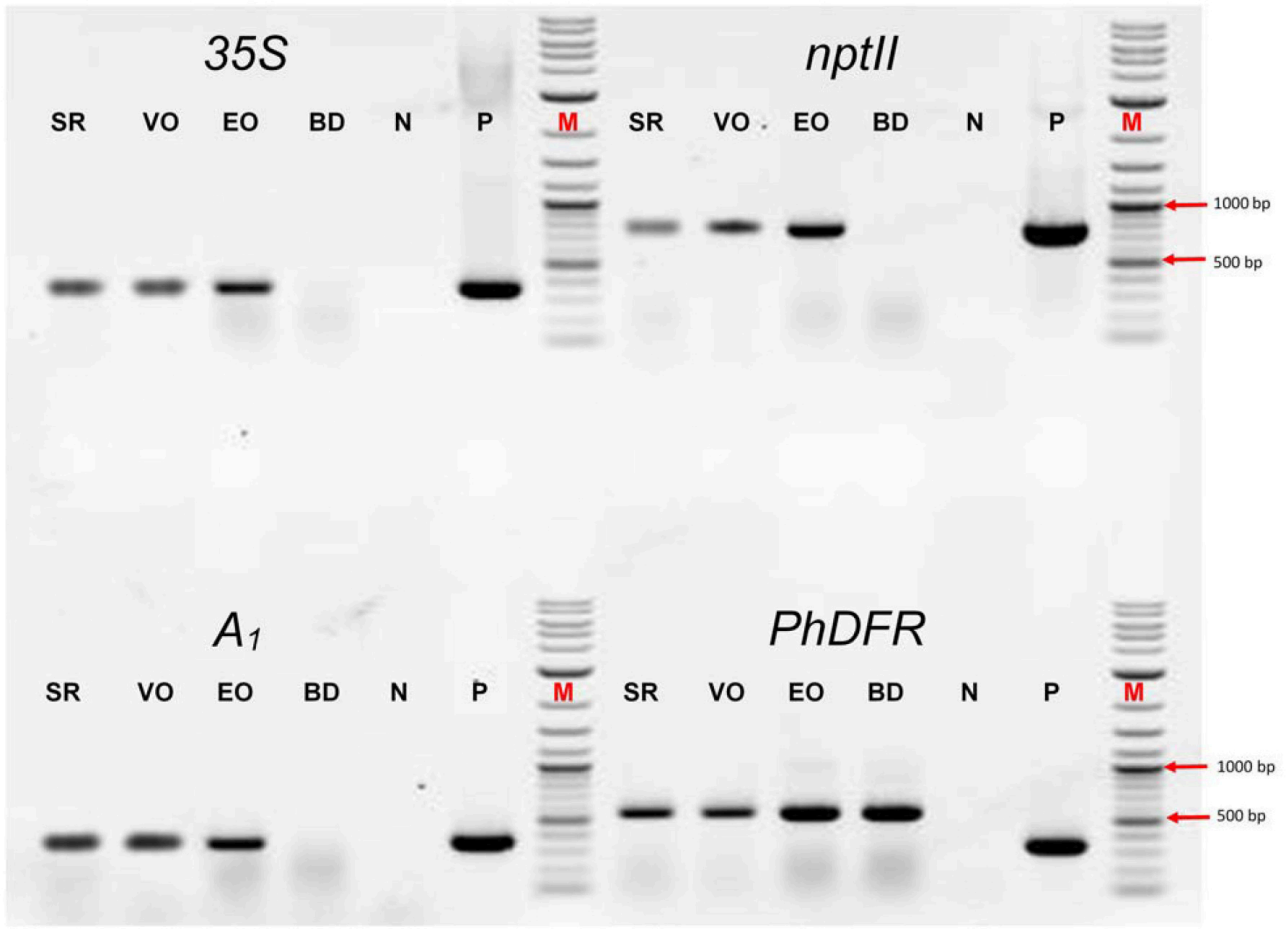
PCR evaluation of genomic DNA of the three orange cultivars Salmon Ray (SR), Viva Orange (VO), and Electric Orange (EO) and the pink-white cultivar BabyDoll (BD) for the presence of specific DNA sequences. Water was used as negative control (N), and plasmids harboring the genes of interest as positive control (P) instead of genomic DNA. M: DNA marker 2-Log DNA Ladder; 1,000 and 500 bp fragments are marked with red arrows. *35S*: partial promoter sequence of the *35S* Cauliflower mosaic virus gene; *nptII*: partial coding sequence of the *nptII* selectable marker gene; *A*_*1*_: partial coding sequence of the *A*_*1*_ gene; *PhDFR*: partial coding sequence of the DFR of *Petunia* × *hybrida*. Primer sequences are provided in Supplementary Table S1. For genomic DNA, fragments of 365 bp (*35S*), 779 bp (*nptII*), 346 bp (*A*_*1*_), and 565 bp (*PhDFR*) are expected. For *35S, nptII* and *A*_*1*_, expected fragment sizes are identical for control plasmid. For *PhDFR*, a fragment of 337 bp for control plasmid DNA (cDNA clone) instead of 565 bp (genomic DNA) is expected.

**Figure 4:**
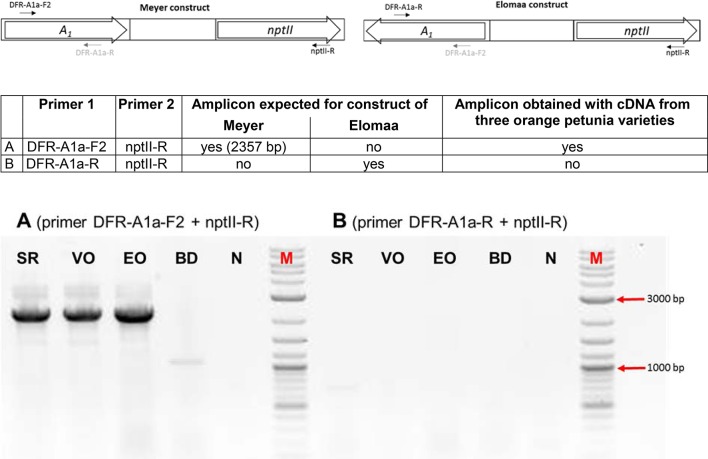
PCR discrimination between the transformation plasmids of Meyer et al. (*A1* and *nptII* cloned in the sense orientation) and Elomaa et al. (*A1* and *nptII* cloned in the antisense direction). PCRs were performed with primer combinations **(A)** (primer DFR-*A1*a-F2 + *nptII*-R) and **(B)** (primer DFR-*A1*a-R + *nptII*-R) with genomic DNA of cvs. Salmon Ray (SR), Viva Orange (VO), Electric Orange (EO), and BabyDoll (BD). N, negative control in which genomic DNA is replaced by water; M, DNA marker 2-Log DNA Ladder; 3,000 and 1,000 bp fragments are marked with red arrows.

We screened the varieties by PCR for sequences that would indicate the presence of a biotechnological construct. With specific primers for *A*_*1*_, *nptII* and the *35S* promoter, PCR fragments could be amplified from genomic DNA of all three varieties (Figure 3), thereby confirming a genetic modification event. To illuminate the origin of the *A*_*1*_source, we analyzed the transgene present in the three orange varieties. Two *A*_*1*_ constructs previously used to create orange GE-lines (Meyer et al., 1986; Elomaa et al., 1995) for scientific purposes are the most probable sources. Discrimination by PCR between these is possible (Table in Figure 4), based on the direction of the *A*_*1*_ gene, which was either sense (Meyer et al., 1986) or antisense (Elomaa et al., 1995) to the *nptII* gene. All three orange petunia varieties showed an approximately 2.3 kb amplicon when PCR with genomic DNA as template and *A*_*1*_ specific forward and *nptII* specific reverse primers was performed (Figure 4), as expected only for the construct of Meyer et al. (1986). We furthermore sequenced a 3.3 kb PCR product obtained from genomic DNA of the three orange lines as templates using a *35S* promoter specific forward and an *OCS terminator* specific reverse primer.

All three amplicons showed identical sequences (Supplementary Figure S1) at the nucleotide level (NCBI MF521566). This included a partial sequence of the *35S* promoter (217 bp), followed by the restriction sites of *Xba*I and *Eco*RI, a 5′ untranslated region (UTR) of the *A*_*1*_ cDNA clone of maize, the 1,074 bp full size *A*_*1*_ cDNA clone, and an adjacent 3′ UTR with a 144 bp insertion consisting of the partial *Cin4-1* transposable element with a polyA stretch as present in the type 2 allele of *A*_*1*_ described by Schwarz-Sommer et al. (1987a,b). After the polyA stretch, we identified an *Eco*RI and *Xba*I restriction site, a 226 bp t*35S* terminator flanked by an *Eco*RI restriction site, and a selection gene cassette including the p*NOS* promoter, *nptII* cDNA clone and t*OCS* terminator (1,402 bp fragment with only partial t*OCS*).

### DFR substrate specificity

Protein preparations obtained from the three orange petunia varieties were, surprisingly, not able to convert DHK under *in vitro* assay conditions, although they showed DFR activity with dihydromyricetin (DHM) as a substrate (Table 2). DHK conversion with enzyme preparation of the orange petunia petals could be observed neither in buds nor in petals. DHQ was not accepted either, with exception of the enzyme preparations from cv. Electric Orange, which converted DHQ to some extent, although at dramatically lower level than DHM (Table 2). Common blue and red petunias, which were used as controls, showed dihydroflavonol conversion levels that were almost comparable to those of the GE-petunia. High conversion rates were obtained with DHM as substrate, whereas DHK was not converted (Table 2). Conversion of DHQ was much lower than with DHM and lower than those observed with preparations from cv. Electric Orange. To exclude a false negative result, the integrity of DHK was confirmed with enzyme preparations from strawberry (Table 2) which possess a DFR showing high DHK substrate specificity (Miosic et al., 2014). Thus, the orange petunias showed the same lack of DHK acceptance under common DFR assay conditions as the blue and red flowering non-GE controls.

**Table 2:** Specific DFR activity with DHK, DHQ, and DHM as substrate using enzyme preparations from petals of petunia cultivars and with recombinant DFR from maize and petunia.

		**Specific activity [nmol/g protein] measured with substrate**		
**Enzyme source**	***A*_*1*_ present**	**DHK**	**DHQ**	**DHM**
Salmon Ray buds/open flowers	Yes	−/−^*^	−/−	1.96/1.94
Electric Orange buds/open flowers	Yes	−/−^*^	0.13/0.12	1.96/1.96
Viva Orange buds/open flowers	Yes	−/−^*^	−/−	1.96/1.96
BabyDoll	No	−/−^*^	−/−	1.96/1.93
Corso Rot	No	−/−^*^	0.01/−	1.96/1.94
Corso Blau	No	−/−^*^	0.03/−	1.96/1.96
Control reaction (strawberry)	No	1.96	0.25	0
recombinant A_1_ protein		–	0.33	0.55
Recombinant wild type *Ph*DFR (137L, 138D)^**^		–	–	1.47
Recombinant *Ph*DFR mutant (137V, 138D)^**^		–	–	1.52
Recombinant *Ph*DFR mutant (137V, 138N)^**^			1.41	1.14
Recombinant *Ph*DFR mutant (137L, 138N)^**^		–	1.48	1.48

*Enzyme preparations from strawberry fruits were used as control to demonstrate integrity of the DHK and DHQ substrates*.

* DHK conversion was not observed even if incubation time was extended to 24 h at 4°C;

** *Numbering according to the deduced amino acid sequence of A_1_ as shown in Supplementary Figure S2; –: below detection level*.

To shed further light on the substrate specificity of the maize DFR encoded by *A*_*1*_, we heterologously expressed an *A*_*1*_ cDNA clone as GST-fusion protein in *E. coli*. After removal of the GST-tag, the purified recombinant A_1_ protein showed high substrate specificity, converting DHQ and DHM to a comparable extent, but no conversion of DHK could be observed during time spans sufficient to exhaust DHQ and DHM (Table 2). Even when incubation times with DHK were extended over night or up to 24 h at 4°C, no conversion of DHK to leucopelargonidin could be observed *in vitro*. Kinetic data indicate a comparable substrate specificity for DHM (*K*_*m*_ 3.1 μM, V_*max*_ 1.0 × 10^−3^ μM/s; *k*_*cat*_ 0.87 s^−1^; *k*_*cat*_*/K*_*m*_ 0.28 s^−1^μM^−1^) and DHQ (*K*_*m*_ 2.9 μM, V_*max*_ 1.25 × 10^−3^ μM/s; *k*_*cat*_ 0.70 s^−1^; *k*_*cat*_*/K*_*m*_ 0.24 s^−1^μM^−1^).

To compare the substrate specificities of the DFRs of maize and *Petunia* × *hybrida*, studies were also performed with recombinant *Petunia* × *hybrida* DFR (*Ph*DFR). Purified recombinant, *Ph*DFR showed no DHQ or DHK conversion and thus, a clear specificity for DHM (Table 2), which was also confirmed by the kinetic data (*K*_*m*_ 1.3 μM; V_*max*_ 0.5 × 10^−3^ μM/s; *k*_*cat*_*/K*_*m*_ 2.14 s^−1^; *k*_*cat*_*/K*_*m*_ 1.6 s^−1^ μM^−1^). Amino acid sequence identity between *Ph*DFR and A_1_ is only 54%. Particularly the region presumably determining substrate specificity shows a difference in positions 132–134 (amino acid numbering according *Ph*DFR). To investigate whether the striking difference in the DHQ acceptance between the two recombinant DFRs is based on this region as suggested (Johnson et al., 2001), we created mutants with altered amino acids (Table 2). Whereas an exchange of the leucine with a valine in position 137 did not result in increased DHQ conversion, the exchange of aspartic acid with asparagine in position 138 raised DHQ conversion from zero to levels equaling those of DHM.

### Gene expression in the orange petunia varieties

The transcriptomes of orange (cv. Salmon Ray), red (cv. Corso Rot) and black (cv. Blackberry) petunias were analyzed for differences in their gene expression pattern with respect to the phenotype. We particularly focused on the genes involved in color formation (Bombarely et al., 2016). The three varieties did not provide a uniform picture (Supplementary Figure S3). Whereas cv. Salmon Ray showed lower gene expressions in many of the structural genes of the flavonoid pathway, but not of the phenylpropanoid pathway, most of the structural genes in the early and late flavonoid pathway seemed to be up-regulated in the two other varieties. However, an increased *DFR:FLS* expression ratio was observed in the red and in all three orange cultivars compared to the black. This was further examined by quantitative real-time PCR.

Quantitative real-time PCR performed with primers discriminating between the DFRs from maize (*A*_*1*_) and petunia (*PhDFR*) (Supplementary Table S1), showed that *PhDFR* expression strongly dominated over *A*_*1*_expression (Figure 2, bar charts) in developing buds of the orange varieties, despite being under the control of the strong, constitutive p*35S* promoter. *A*_1_*/PhDFR* expression ratios of approximately 1 could only be observed in open flowers. The qPCR studies also confirmed that the three orange petunia varieties had a low expression of *F3'H* and *FLS* and thus a very high *DFR:FLS* expression ratio during the flower life cycle (**Figure 6**). There was, however, no statistically significant correlation between the *A*_1_*/PhDFR* expression ratios and the pelargonidin-type anthocyanidin concentration in the petals (Figure 2).

## Discussion

The DFR of petunia has ever been the role model for studies on DFR substrate specificity, and the resulting lack of orange flower color in petunia has always been the best example for the complex mechanisms of color establishment in flowers. The creation of an orange petunia by a transgenic approach in the 1980s at the Max Planck Institute for Plant Breeding Research in Cologne, Germany was a further landmark in the field of flower color research and the subsequent field trial attracted attention far beyond the horticultural community. Thus, the fact that petunia does not naturally possess orange flower color, and the underlying biochemical reason, has been established knowledge for a few decades (Meyer et al., 1986, 1992; Johnson et al., 1999, 2001). Therefore, the appearance of orange petunia varieties on the European market attracted the interest of scientists familiar with anthocyanin flower color. As they were not declared as genetically modified plants, which would have been compulsory if a transgenic breeding method had been used, they were apparently a result of classical breeding. Recent research demonstrated, however, that the vast majority of the commercially available orange petunia varieties, but not all, are genetically engineered and harbor the *A*_*1*_ cDNA clone from *Zea mays* (Bashandy and Teeri, 2017). Despite the general consensus that the transgenic construct most probably derived from the first scientific GE-petunia (Meyer et al., 1986), it always remained unmentioned that there was a second scientific petunia (Elomaa et al., 1995), which was constructed with the same GE-elements, which could have been a possible source of the putatively unintentionally escaped *A*_*1*_ cDNA clone. Our data unequivocally demonstrate, however, that of these two possible known sources, an unintentional release of the construct of the Elomaa et al. (1995) can indeed be excluded.

In an independent approach we analyzed three varieties released by breeders from three countries, The Netherlands, Israel and Germany. All three amplicons showed identical sequences (Supplementary Figure S1) at the nucleotide level (NCBI MF521566), demonstrating that a single *A*_*1*_ source had entered breeding programmes worldwide. The sequence we obtained was in line with NCBI KY964325 (Bashandy and Teeri, 2017). There is, however, an overlap, as our primers started 699 bp downstream at the 5′-end, but provided an additional 375 bp stretch at the 3′-end.

**Figure 5:**
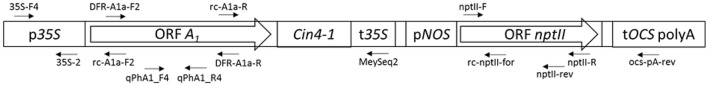
Schematic representation of the transgenic insert found in the three GE-petunia varieties cvs. Salmon Ray, Viva Orange and Electric Orange (NCBI MF521566) p*35S*, promoter sequence of the *35S* Cauliflower mosaic virus gene; *A*_*1*_, coding sequence of the *A*_*1*_ gene; *Cin4-1*, partial *Cin4-1* transposable element present in type 2 allele of *A*_*1*_ according to Schwarz-Sommer et al. (1987a,b); t*35S*, terminator sequence of the *35S* Cauliflower mosaic virus gene; p*NOS*, promoter sequence of the nopaline synthase gene; *nptII*, coding sequence of the neomycine phosphotransferase II selectable marker gene; t*OCS*, terminator sequence of the octopine synthase gene. Positions of primers are marked by arrows. Primer sequences are provided in Supplementary Table S1.

Our sequencing results identified with near absolute certainty the transformation construct of Meyer et al. (1986) as the *A*_*1*_ source, based on the following characteristics (Figure 5, Supplementary Figure S1): the same arrangement of *35S* promoter, *A*_1_*, 35S* terminator*, nopaline synthase* (*NOS*) promoter, *nptII, octopine synthase (OCS)* terminator, and the restriction sites used for the p35A1 plasmid construction, as described for the Meyer et al. (1986) construct, was identified (Figure 5). Pre-eminently, the transgene found in the three orange petunia varieties contains the *A*_*1*_ type 2 allele previously used for plasmid p35A1 construction (Meyer et al., 1986), which includes, at the 3′-end, an additional segment from the non-viral transposable *Cin4-1* sequence (Schwarz-Sommer et al., 1987a). In addition, in the transition zone between the *35S* promoter and the *A*_*1*_ gene, parts of the untranslated sequences of the 5′ flanking region, described previously (Schwarz-Sommer et al., 1987b), are present. This rules out other potential, as yet unidentified, sources, as it is unlikely that a putative third, yet unknown construct would harbor this special *A*_*1*_ allele, particularly as the transposable element does not add any functional advantage with respect to color formation.

Unexpectedly, the orange petunias showed the same lack of DHK acceptance under common DFR assay conditions as the blue and red flowering non-GE controls. Concordantly, the orange petals accumulate large amounts of DHK derivatives (Table 1) that have not been ultimately converted to pelargonidin-based pigments. Such elevated dihydroflavonol levels were not found in common petunia varieties with a high F3′5′H activity, as this favors creation of delphinidin based pigments. Likewise, in the absence of F3′5′H activity, accumulated DHK and DHQ can be converted to flavonols, if FLS is active in the petals, as is the case in the red variety (Table 1). The low substrate specificity for DHK was surprising, given the fact that *A*_*1*_ had been introduced to explicitly enable conversion of DHK and thus, formation of pelargonidin based pigments. Much better color effects had been achieved by transformation with an unspecific gerbera *DFR* (Elomaa et al., 1995), thereby already pointing to a low substrate specificity of the maize DFR for DHK, although observed effects were rather attributed to the instability of monocotyledonous cDNA in the dicotyledonous petunia (Elomaa et al., 1995). The low substrate specificity of the protein encoded by the *A*_*1*_ gene was confirmed with recombinant maize DFR obtained by heterologous expression in *E. coli*.

Only low expression rates were observed for the *A*_*1*_ gene, which is in accordance with findings of epigenetic downregulation effects in GE-petunia (Linn et al., 1990; Meyer and Heidmann, 1994; Meyer, 1998). *A*_*1*_ gene expression generally remained below the rates measured for the *PhDFR*. Highest expression was found in fully developed flowers, where an *A*_1_*/PhDFR* ratio of up to 1 could be measured. Despite this, the content of pelargonidin-based pigments remained surprisingly unchanged during flower development (Figure 2). There was, however, no statistically relevant correlation between the pelargonidin-based pigment concentration and the *A*_*1*_ gene expression. Moreover, we never observed DHK conversion with enzyme preparations of the orange petunia petals independently of a high (open flowers) or low *A*_1_*/PhDFR* (buds) expression ratio, raising the question, if, besides the maize DFR, the petunia DFR could also contribute to pelargonidin-precursor production.

**Figure 6:**
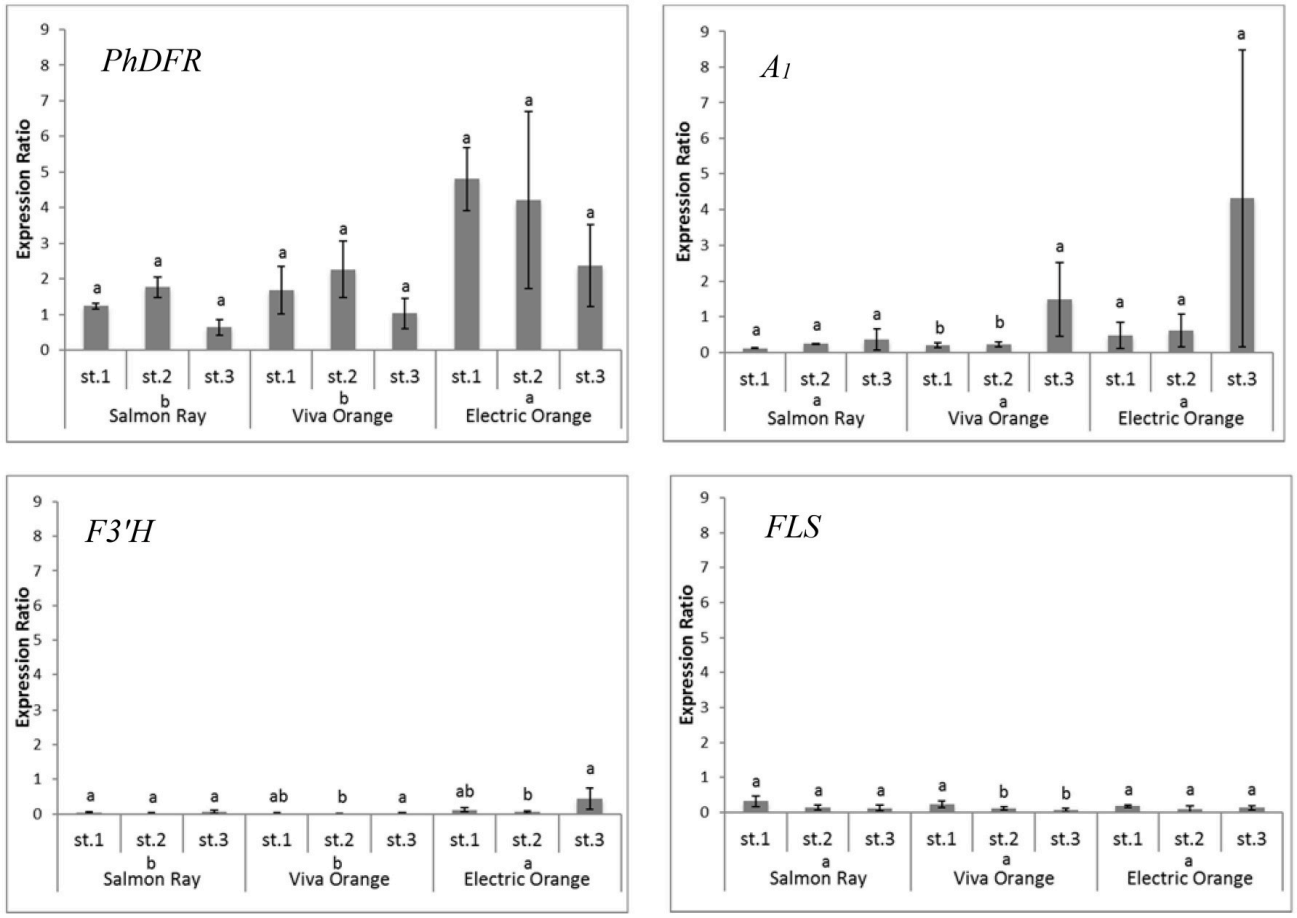
Quantitative gene expression of *PhDFR, A*_*1*_, *F3*′*H* and *FLS*, normalized to *actin* in three developmental stages (st. 1: buds of 0.6–3 cm length, st. 2: buds of 3–5 cm length, st. 3: open flowers) of orange petunia flowers in three varieties (cvs. Salmon Ray, Viva Orange, Electric Orange). Average values were calculated from at least three biological replications collected at different sites. Error bars show standard deviation. Different letters above bars denote statistical difference according to Duncan test (*p* < 0.05) between the developmental stages separately for each variety. Different letters above cultivar names denote statistical difference according to Duncan test (*p* < 0.05) between the three varieties in general calculated from pooled stages.

To determine how the orange coloration may occur at all, if a poorly expressed non-petunia *DFR*, with an additionally low substrate specificity for DHK, was present in GE-petunia petals, we compared the transcriptomes of the three orange varieties (cvs. Salmon Ray, Viva Orange, Electric Orange), red (cv. Corso Rot) and black (cv. Blackberry) petunias (Supplementary Figure S3) and analyzed the genes particularly involved in color formation (Bombarely et al., 2016). An increased *DFR:FLS* expression ratio in the red and orange cultivars was observed, compared to the black, which was confirmed by qPCR (Figure 6) and by the relatively small amounts of flavonols found in the petals of orange varieties (Table 1).

Apparently, the establishment of orange petunia flower color can occur only in the absence of interfering F3′H and FLS activities (Figure 6). Considering the low substrate specificity of both *Ph*DFR and A_1_ protein for DHK, sufficient leucopelargonidin precursor for pelargonidin-type flowers will be synthesized (i) very slowly over a long time, (ii) only if no other dihydroflavonol precursor is present (absent or low F3′H and F3′5′H activity), and (iii) if the accumulating DHK is not redirected by a highly active FLS toward flavonols. In the same way, cyanidin-based red flowering petunia varieties occur naturally, despite the fact that *Ph*DFR shows stringent specificity for DHM as substrate (Table 2). The red cultivar used as a control accumulates a substantial amount of dihydroflavonols, however, thereby confirming the low *FLS* expression and elevated *DFR:FLS* expression ratio in the red cultivars already indicated by the transcriptome analysis. Considering the DFR substrate specificity of petunia for DHM, increased flavonol formation, at the expense of anthocyanin accumulation, and thus, only a pale color, would be expected in the case of a highly active petunia FLS. The elaborate creation of transgenic petunia and the current global commotion surrounding the escaped *A*_*1*_ gene seem to be a great cause, in comparison to the relatively small color effects attained by the use of the inefficient maize DFR.

American and European authorities unambiguously stated that the GE-petunia is not harmful to consumers and environment. It is still unknown, how plants harboring the *A*_*1*_ construct of Meyer et al. (1986) entered classical breeding programmes. Nefarious use of GE-plants is unlikely, due to foreseeable troubles when plants inevitably attract attention. There are, however, several scenarios how the GE-petunia could have escaped. After its creation at the Max Planck Institute for Plant Breeding Research in Cologne (Meyer et al., 1986), and the contentious field trial in Germany in 1990, the plants were kept in several institutions, and were also used for breeding purposes (Oud et al., 1995; Servick, 2017), followed by field trials in the US. Currently, the most favored explanation (Servick, 2017) seems to be that during a chain of company fusions the GE-background of orange petunias was forgotten, and the lines could therefore enter new breeding programmes. Our results demonstrate why the presence of the *A*_*1*_ does not result in orange phenotypes in a common biochemical petunia background, which facilitates undetected dispersion. Thus, the original GE-petunia (Meyer et al., 1986) or progenies thereof, created by classical breeding (Oud et al., 1995) or—less likely—by escaped pollen, in the field or in the greenhouse, could have infiltrated classical breeding chains, unless it re-emerged as orange petunia in a rare event of a proper genetic background. The large spectrum of undeclared GE-petunia varieties can be explained by the use of early orange varieties as parent plants in classical breeding attempts for further orange varieties by other companies and by the use of non-orange breeding material harboring an unrecognized *A*_*1*_. The fact that a single construct was found so far in the orange petunia varieties, as opposed to plural different constructs, points at a single event in the breeding chain rather than at multiple parallel events.

In the current debate, it was iterated that real orange petunia flower colors cannot occur naturally (David, 2017). Some few petunia varieties, however, show a pattern of red and yellow pigments that, at a glance, might be mistaken for orange (Bashandy and Teeri, 2017). But even minor mutations in the active site of the DFR can result in higher DHK specificity, as demonstrated by the existing patent for a DHK specific DFR (Johnson et al., 2001). A spontaneous mutation occurring in a suitable background, although unlikely, could indeed provide a naturally orange-flowering petunia. This could also be achieved by cutting-edge genome editing methods causing a targeted mutation, which can currently not be distinguished from mutations induced by well accepted methods such as mutation via chemicals or radiation. It remains to be seen how the escaped GE-petunias will influence the current debate about the classification of genome editing as a genetic engineering method, and on biotech patents in general, which was provoked by the recent barley patents obtained by large brewing companies.

## Availability of data and materials

All data supporting the findings is contained in the manuscript and its supplementary files. Transcriptome data are available at the Short Read Archive of the International Nucleotide Sequence Database Collaboration: SAMN07988829 (*Petunia* × *hybrida* cv. Blackberry), SAMN07988830 (*Petunia* × *hybrida* cv. Corso Rot), SAMN07988831 (*Petunia* × *hybrida* cv. Viva Orange), SAMN07988832 (*Petunia* × *hybrida* cv. Electric Orange), SAMN07988833 (*Petunia* × *hybrida* cv. Salmon Ray).

## Author contributions

HH, CH-G, and KS: Conceived the research and wrote the manuscript; SM, DN, BR, BW, RP, RL, and LE: conducted the experiments; TR and KO: analyzed the data. All authors approved the manuscript.

### Conflict of interest statement

The authors declare that the research was conducted in the absence of any commercial or financial relationships that could be construed as a potential conflict of interest.
