# Supplementary material for: Great Cause—Small Effect: Undeclared Genetically Engineered Orange Petunias Harbor an Inefficient Dihydroflavonol 4-Reductase
Source: Front Plant Sci. 2018 Feb 28;9:149. doi: 10.3389/fpls.2018.00149 (PMC5835687; doi:10.3389/fpls.2018.00149)
Supplement: Supplementary file 1 [file DataSheet1.docx]

**Supplementary Table S1: Primers used for molecular evaluation of the GE-petunias.**If applicable, their position in the construct is shown in Fig. 5.

| **Gene** | **Primer: sequence (5’-3’)** | **Annealing** | **Purpose** | **Amplicon size (bp)** | | |
| --- | --- | --- | --- | --- | --- | --- |
|  |  | **T (°C)** |  | gDNA | cDNA |  |
| p*35S* | 35S-F4: CATGGTGGAGCACGACACTC  35S-2 GATAGTGGGATTGTGCGTCA | 60 | transgene detection, sequencing | 365 | - |  |
| *nptII* | nptII-rev: atcgggagcggcgataccgta  rc-nptII-for CAGTCATAGCCGAATAGCCTC  nptII-F: CAAGATGGATTGCACGCAGG nptII-R: AACTCGTCAAGAAGGCGATAG | -  -  61 | sequencing  sequencing  transgene detection | -  -  779 | -  -  779 |  |
| *A_1_* | DFR-A1a-F2: GGGCATGCAAGGAGGCCGG  DFR-A1a-R: GAGGTCGTCGAGGTGGATGAGC  qPhA1_F4 CGACTTCTGCCGTCGCG  qPhA1_R4 GATGATGGTGACCAGGTCCAG  rc-A1a-R GCTCATCCACCTCGACGACCTC  rc-A1a-F2 CCGGCCTCCTTGCATGCCC | 65  72  -  - | transgene detection  quantitative PCR*  sequencing  sequencing | 346  -  -  - | 346  118  -  - |  |
| t*35S* | MeySeq2: AACACATGAGCGAAACCCTATA | - | sequencing | - | - |  |
| *PhDFR* | Pet-DFR-F1: TCACTTCATCTGCTGGAACTCTCGATG  Pet-DFR-R: GCCTCACAAAGATCATCCAAATGCACATAT  qPhDFR_F: CCTTCCCGGAGCAACGG  qPhD_R: TGGCGACGTGGAAGACG | 65  72 | transgene detection  quantitative PCR* | 565  - | 337  94 |  |
| t*OCS* | ocs-pA-rev: GTAACGGGTGATATATTCATTAGAATG | - | sequencing | - | - |  |
| *Actin* | PetAct11_qF: TGCACTCCCACATGCTATCCT  PetAct11_qR: TCAGCCGAAGTGGTGAAAGAG | 72 | quantitative PCR* | - | 114 |  |
| *FLS* | PetFLSq_F: GATGAGGCTATCGCGGATTTAC  PetFLSq_R: GTCGTTTGATCCTGGAGTCTTG | 72 | quantitative PCR* | - | 102 |  |
| *F3*’*H* | PHT1F3H_qF CTCATTTCTTCTACAATTCATTCTTAGATC  PHT1F3H_qF GGTCCAAGATGGACTAGGTTTC | 72 | quantitative PCR* | - | 114 |  |

*amplification efficiencies: *A_1_* (2.00), *Actin* (2.04), *PhDFR* (2.05), *FLS* (2.02), *F3*’*H* (2.00)

**Supplementary Table S2: List of primers used for subcloning *DFR*s into the bacterial expression vector pGEX-6P-1 and site-directed mutagenesis**

| **Primer Name** | **Sequences (5´>3´- direction)** | **T_m_ (°C)** |
| --- | --- | --- |
| A_1_DFR-FL | GATCCATGGAGAGAGGTGCCGGT | 58.0 |
| A_1_DFR-FS | CATGGAGAGAGGTGCCGGT | 58.0 |
| A_1_DFR-RL | AATTCTTAAGCGCCAATCGTCG | 56.0 |
| A_1_DFR-RS | CTTAAGCGCCAATCGTCG | 56.0 |
| PhDFR-FL | GATCCATGCCCCTTCACCTCCG | 58.0 |
| PhDFR-FS | CATGCCCCTTCACCTCCG | 58.0 |
| PhDFR-RL | AATTCCTAGACTTCAACATTGCTTAACATTTCTG | 61.0 |
| PhDFR-RS | CCTAGACTTCAACATTGCTTAACATTTCTG | 61.0 |
| Ph_DFR_135V_f | TGCTGGAACTGTCGATGTGCA | 65.0 |
| Ph_DFR_135V_r | GATGAAGTGAAAACCAGCC | 60.0 |
| Ph_DFR_136N_f | TGGAACTCTCAATGTGCAAGAG | 59.0 |
| Ph_DFR_136N_r | GCAGATGAAGTGAAAACC | 57.0 |
| Ph_DFR_135NV_f | tgtgCAAGAGCAACAAAAACTTTTC | 57.0 |
| Ph_DFR_135NV_r | ttgacAGTTCCAGCAGATGAAGTG | 61.0 |

**Supplementary Figure S1: Sequence alignment of the 3.3 kb transgene obtained from three orange petunia cultivars (1: Salmon Ray; 2: Viva Orange, 3: Electric Orange).**
p*35S*, partial promoter sequence of the *35S* Cauliflower mosaic virus gene; 5’UTR, 5’ untranslated region according to Schwarz-Sommer et al. (1987a,b), *A_1_*, coding sequence of the *A_1_* gene; 3’UTR, 3’ untranslated region according to Schwarz-Sommer et al. (1987a,b), ISE, insertion site of elements identified in mutable *A_1_* alleles (Schwarz-Sommer et al., 1987a,b), *Cin4-1*, partial *Cin4-1* transposable element present in type 2 allele of *A_1_* (Schwarz-Sommer et al. 1987a,b); t*35S*, terminator sequence of the *35S* Cauliflower mosaic virus gene; p*NOS*, promoter sequence of the nopaline synthase gene; *nptII*, coding sequence of the neomycine phosphotransferase II selectable marker gene; t*OCS*, partial terminator sequence of the octopine synthase gene. For better visualization, every second functional DNA segment (p*35S, A_1_, ISE,* t*35S,* p*NOS,* t*OCS*) is highlighted with grey background. Restriction enzyme sites are boxed.

partial p*35S*

1 50

1 AGAT GGACCCCCAC CCACGAGGAG

2 GATGCCTCTG CCGACAGTGG TCCCAAAGAT GGACCCCCAC CCACGAGGAG

3 GATGCCTCTG CCGACAGTGG TCCCAAAGAT GGACCCCCAC CCACGAGGAG

Partial p*35S*

51 100

1 CATCGTGGAA AAAGAAGACG TTCCAACCAC GTCTTCAAAG CAAGTGGATT

2 CATCGTGGAA AAAGAAGACG TTCCAACCAC GTCTTCAAAG CAAGTGGATT

3 CATCGTGGAA AAAGAAGACG TTCCAACCAC GTCTTCAAAG CAAGTGGATT

Partial p*35S*

101 150

1 GATGTGATAT CTCCACTGAC GTAAGGGATG ACGCACAATC CCACTATCCT

2 GATGTGATAT CTCCACTGAC GTAAGGGATG ACGCACAATC CCACTATCCT

3 GATGTGATAT CTCCACTGAC GTAAGGGATG ACGCACAATC CCACTATCCT

partial p*35S*

151 200

1 TCGCAAGACC CTTCCTCTAT ATAAGGAAGT TCATTTCATT TGGAGAGGAC

2 TCGCAAGACC CTTCCTCTAT ATAAGGAAGT TCATTTCATT TGGAGAGGAC

3 TCGCAAGACC CTTCCTCTAT ATAAGGAAGT TCATTTCATT TGGAGAGGAC

*Eco*RI

partial p*35S*

5’UTR

*Xba*l

201 217 233 250

1 AGGGTACCCG GGGATCCTCT AGAGAATTCC AGCTGCTCAC TCAGTCCTGC

2 AGGGTACCCG GGGATCCTCT AGAGAATTCC AGCTGCTCAC TCAGTCCTGC

3 AGGGTACCCG GGGATCCTCT AGAGAATTCC AGCTGCTCAC TCAGTCCTGC

*A_1_*

5’UTRR

251 292 300

1 GCAAGAGCTC GCTCTCGGAG AAAAAAACGC GGGAGGCGAT AATGGAGGGA

2 GCAAGAGCTC GCTCTCGGAG AAAAAAACGC GGGAGGCGAT AATGGAGGGA

3 GCAAGAGCTC GCTCTCGGAG AAAAAAACGC GGGAGGCGAT AATGGAGGGA

*A_1_*

301 350

1 GGTGCCGGTG CGAGCGAGAA AGGGACGGTG CTGGTCACGG GGGCGTCGGG

2 GGTGCCGGTG CGAGCGAGAA AGGGACGGTG CTGGTCACGG GGGCGTCGGG

3 GGTGCCGGTG CGAGCGAGAA AGGGACGGTG CTGGTCACGG GGGCGTCGGG

*A_1_*

351 400

1 CTTCGCCGGC TCCTGGCTCG TCATGAAGCT CCTCCAGGCC GGCTACACCG

2 CTTCGCCGGC TCCTGGCTCG TCATGAAGCT CCTCCAGGCC GGCTACACCG

3 CTTCGCCGGC TCCTGGCTCG TCATGAAGCT CCTCCAGGCC GGCTACACCG

*A_1_*

401 450

1 TCCGGGCGAC CGTGCGCGAT CCCGCGAACG TTGGGAAGAC GAAGCCATTG

2 TCCGGGCGAC CGTGCGCGAT CCCGCGAACG TTGGGAAGAC GAAGCCATTG

3 TCCGGGCGAC CGTGCGCGAT CCCGCGAACG TTGGGAAGAC GAAGCCATTG

*A_1_*

451 500

1 ATGGACCTTC CCGGAGCAAC GGAGCGCCTG TCCATATGGA AAGCCGACCT

2 ATGGACCTTC CCGGAGCAAC GGAGCGCCTG TCCATATGGA AAGCCGACCT

3 ATGGACCTTC CCGGAGCAAC GGAGCGCCTG TCCATATGGA AAGCCGACCT

*A_1_*

501 550

1 GGCGGAGGAA GGCAGCTTCC ACGACGCCAT CAGGGGCTGC ACCGGCGTCT

2 GGCGGAGGAA GGCAGCTTCC ACGACGCCAT CAGGGGCTGC ACCGGCGTCT

3 GGCGGAGGAA GGCAGCTTCC ACGACGCCAT CAGGGGCTGC ACCGGCGTCT

*A_1_*

551 600

1 TCCACGTCGC CACGCCCATG GACTTCCTGT CCAAAGACCC TGAGAATGAG

2 TCCACGTCGC CACGCCCATG GACTTCCTGT CCAAAGACCC TGAGAATGAG

3 TCCACGTCGC CACGCCCATG GACTTCCTGT CCAAAGACCC TGAGAATGAG

*A_1_*

601 650

1 GTAATCAAGC CGACGGTGGA AGGGATGATA AGCATCATGC GGGCATGCAA

2 GTAATCAAGC CGACGGTGGA AGGGATGATA AGCATCATGC GGGCATGCAA

3 GTAATCAAGC CGACGGTGGA AGGGATGATA AGCATCATGC GGGCATGCAA

*A_1_*

651 700

1 GGAGGCCGGC ACCGTGCGGC GCATCGTCTT CACTTCCTCC GCCGGGACGG

2 GGAGGCCGGC ACCGTGCGGC GCATCGTCTT CACTTCCTCC GCCGGGACGG

3 GGAGGCCGGC ACCGTGCGGC GCATCGTCTT CACTTCCTCC GCCGGGACGG

*A_1_*

701 750

1 TCAACCTGGA GGAGCGGCAG AGGCCCGTCT ACGACGAGGA AAGCTGGACC

2 TCAACCTGGA GGAGCGGCAG AGGCCCGTCT ACGACGAGGA AAGCTGGACC

3 TCAACCTGGA GGAGCGGCAG AGGCCCGTCT ACGACGAGGA AAGCTGGACC

*A_1_*

751 800

1 GACGTCGACT TCTGCCGTCG CGTCAAGATG ACAGGATGGA TGTACTTCGT

2 GACGTCGACT TCTGCCGTCG CGTCAAGATG ACAGGATGGA TGTACTTCGT

3 GACGTCGACT TCTGCCGTCG CGTCAAGATG ACAGGATGGA TGTACTTCGT

*A_1_*

801 850

1 GTCTAAAACC CTGGCGGAGA AGGCGGCCCT GGCGTACGCG GCGGAGCACG

2 GTCTAAAACC CTGGCGGAGA AGGCGGCCCT GGCGTACGCG GCGGAGCACG

3 GTCTAAAACC CTGGCGGAGA AGGCGGCCCT GGCGTACGCG GCGGAGCACG

*A_1_*

851 900

1 GCCTGGACCT GGTCACCATC ATCCCGACGC TCGTGGTCGG CCCGTTCATC

2 GCCTGGACCT GGTCACCATC ATCCCGACGC TCGTGGTCGG CCCGTTCATC

3 GCCTGGACCT GGTCACCATC ATCCCGACGC TCGTGGTCGG CCCGTTCATC

*A_1_*

901 950

1 AGCGCGTCCA TGCCGCCCAG CCTCATCACC GCGCTGGCGC TCATCACGGG

2 AGCGCGTCCA TGCCGCCCAG CCTCATCACC GCGCTGGCGC TCATCACGGG

3 AGCGCGTCCA TGCCGCCCAG CCTCATCACC GCGCTGGCGC TCATCACGGG

*A_1_*

951 1000

1 GAACGCGCCG CACTACTCGA TCCTCAAGCA GGTGCAGCTC ATCCACCTCG

2 GAACGCGCCG CACTACTCGA TCCTCAAGCA GGTGCAGCTC ATCCACCTCG

3 GAACGCGCCG CACTACTCGA TCCTCAAGCA GGTGCAGCTC ATCCACCTCG

*A_1_*

1001 1050

1 ACGACCTCTG CGACGCCGAG ATCTTCCTCT TCGAGAACCC GGCCGCGGCC

2 ACGACCTCTG CGACGCCGAG ATCTTCCTCT TCGAGAACCC GGCCGCGGCC

3 ACGACCTCTG CGACGCCGAG ATCTTCCTCT TCGAGAACCC GGCCGCGGCC

*A_1_*

1051 1100

1 GGGCGCTACG TTTGCTCCTC GCACGACGTC ACCATCCACG GCCTCGCCGC

2 GGGCGCTACG TTTGCTCCTC GCACGACGTC ACCATCCACG GCCTCGCCGC

3 GGGCGCTACG TTTGCTCCTC GCACGACGTC ACCATCCACG GCCTCGCCGC

*Kpn*I

*A_1_*

1101 1150

1 CATGCTCAGG GATAGGTACC CCGAGTACGA CGTCCCGCAG AGGTTCCCCG

2 CATGCTCAGG GATAGGTACC CCGAGTACGA CGTCCCGCAG AGGTTCCCCG

3 CATGCTCAGG GATAGGTACC CCGAGTACGA CGTCCCGCAG AGGTTCCCCG

*A_1_*

1151 1200

1 GGATCCAGGA CGACCTCCAG CCGGTGCGCT TCTCGTCCAA GAAGCTCCAG

2 GGATCCAGGA CGACCTCCAG CCGGTGCGCT TCTCGTCCAA GAAGCTCCAG

3 GGATCCAGGA CGACCTCCAG CCGGTGCGCT TCTCGTCCAA GAAGCTCCAG

*A_1_*

1201 1250

1 GACCTCGGGT TCACCTTCAG GTACAAGACG CTGGAGGACA TGTTCGACGC

2 GACCTCGGGT TCACCTTCAG GTACAAGACG CTGGAGGACA TGTTCGACGC

3 GACCTCGGGT TCACCTTCAG GTACAAGACG CTGGAGGACA TGTTCGACGC

*A_1_*

1251 1300

1 CGCCATCCGG ACTTGCCAGG AGAAGGGCCT CATCCCCCTC GCCACTGCCG

2 CGCCATCCGG ACTTGCCAGG AGAAGGGCCT CATCCCCCTC GCCACTGCCG

3 CGCCATCCGG ACTTGCCAGG AGAAGGGCCT CATCCCCCTC GCCACTGCCG

*A_1_*

1301 1350

1 CCGGAGGGGA CGGCTTTGCC TCGGTGCGCG CACCCGGCGA GACGGAGGCG

2 CCGGAGGGGA CGGCTTTGCC TCGGTGCGCG CACCCGGCGA GACGGAGGCG

3 CCGGAGGGGA CGGCTTTGCC TCGGTGCGCG CACCCGGCGA GACGGAGGCG

3’UTR

*A_1_*

1351 1365 1400

1 ACGATTGGCG CTTAGGCAAC GATCCCCCGG CTCTCCCCGT CGATATGATG

2 ACGATTGGCG CTTAGGCAAC GATCCCCCGG CTCTCCCCGT CGATATGATG

3 ACGATTGGCG CTTAGGCAAC GATCCCCCGG CTCTCCCCGT CGATATGATG

ISE

*Cin4-1*

3’UTR

1401 1413 1421 1450

1 CAATCAGCTA TCTATCTCTT GTTTGCCAAA AAAAATAAGG GAGGTCTTGG

2 CAATCAGCTA TCTATCTCTT GTTTGCCAAA AAAAATAAGG GAGGTCTTGG

3 CAATCAGCTA TCTATCTCTT GTTTGCCAAA AAAAATAAGG GAGGTCTTGG

*Cin4-1*

1451 1500

1 CATACTCGAT CTAGAGCGCT TTGCAAGAGC GTTAAGGCTT AGATGACTAT

2 CATACTCGAT CTAGAGCGCT TTGCAAGAGC GTTAAGGCTT AGATGACTAT

3 CATACTCGAT CTAGAGCGCT TTGCAAGAGC GTTAAGGCTT AGATGACTAT

*Cin4-1*

1501 1550

1 GGCTACGATG GACGAATAGA GACAAAGCAT GGACTGGGTT GCAATTAAAA

2 GGCTACGATG GACGAATAGA GACAAAGCAT GGACTGGGTT GCAATTAAAA

3 GGCTACGATG GACGAATAGA GACAAAGCAT GGACTGGGTT GCAATTAAAA

*polyA*

*Eco*RI

t*35S*

*Xba*l

1551 1562 1577 1600

1 AAAAAAAAAA AAAGGAATTC TCTAGAGTCG ACCTGCAGGC ATGCCCGCTG

2 AAAAAAAAAA AAAGGAATTC TCTAGAGTCG ACCTGCAGGC ATGCCCGCTG

3 AAAAAAAAAA AAAGGAATTC TCTAGAGTCG ACCTGCAGGC ATGCCCGCTG

t*35S*

1601 1650

1 AAATCACCAG TCTCTCTCTA CAAATCTATC TCTCTCTATA ATAATGTGTG

2 AAATCACCAG TCTCTCTCTA CAAATCTATC TCTCTCTATA ATAATGTGTG

3 AAATCACCAG TCTCTCTCTA CAAATCTATC TCTCTCTATA ATAATGTGTG

t*35S*

1651 1700

1 AGTAGTTCCC AGATAAGGGA ATTAGGGTTC TTATAGGGTT TCGCTCATGT

2 AGTAGTTCCC AGATAAGGGA ATTAGGGTTC TTATAGGGTT TCGCTCATGT

3 AGTAGTTCCC AGATAAGGGA ATTAGGGTTC TTATAGGGTT TCGCTCATGT

t*35S*

1701 1750

1 GTTGAGCATA TAAGAAACCC TTAGTATGTA TTTGTATTTG TAAAATACTT

2 GTTGAGCATA TAAGAAACCC TTAGTATGTA TTTGTATTTG TAAAATACTT

3 GTTGAGCATA TAAGAAACCC TTAGTATGTA TTTGTATTTG TAAAATACTT

t*35S*

1751 1800

1 CTATCAATAA AATTTCTAAT TCCTAAAACC AAAATCCAGG GGTACCGAGC

2 CTATCAATAA AATTTCTAAT TCCTAAAACC AAAATCCAGG GGTACCGAGC

3 CTATCAATAA AATTTCTAAT TCCTAAAACC AAAATCCAGG GGTACCGAGC

*Eco*RI

1801 1850

1 TCGAATTCTC ACTCATTAGG CACCCCAGGC TTTACACTTT ATGCTTCCGG

2 TCGAATTCTC ACTCATTAGG CACCCCAGGC TTTACACTTT ATGCTTCCGG

3 TCGAATTCTC ACTCATTAGG CACCCCAGGC TTTACACTTT ATGCTTCCGG

1851 1900

1 CTCGTATAAT GTGTGGAATT GTGAGCGGAT AACAATTTCA CACAGGAAAC

2 CTCGTATAAT GTGTGGAATT GTGAGCGGAT AACAATTTCA CACAGGAAAC

3 CTCGTATAAT GTGTGGAATT GTGAGCGGAT AACAATTTCA CACAGGAAAC

p*NOS*

1901 1950

1 AGGATCATGA GCGGAGAATT AAGGGAGTCA CGTTATGACC CCCGCCGATG

2 AGGATCATGA GCGGAGAATT AAGGGAGTCA CGTTATGACC CCCGCCGATG

3 AGGATCATGA GCGGAGAATT AAGGGAGTCA CGTTATGACC CCCGCCGATG

p*NOS*

1951 2000

1 ACGCGGGACA AGCCGTTTTA CGTTTGGAAC TGACAGAACC GCAACGTTGA

2 ACGCGGGACA AGCCGTTTTA CGTTTGGAAC TGACAGAACC GCAACGTTGA

3 ACGCGGGACA AGCCGTTTTA CGTTTGGAAC TGACAGAACC GCAACGTTGA

2001 2050

p*NOS*

1 AGGAGCCACT CAGCCGCGGG TTTCTGGAGT TTAATGAGCT AAGCACATAC

2 AGGAGCCACT CAGCCGCGGG TTTCTGGAGT TTAATGAGCT AAGCACATAC

3 AGGAGCCACT CAGCCGCGGG TTTCTGGAGT TTAATGAGCT AAGCACATAC

p*NOS*

2051 2100

1 GTCAGAAACC ATTATTGCGC GTTCAAAAGT CGCCTAAGGT CACTATCAGC

2 GTCAGAAACC ATTATTGCGC GTTCAAAAGT CGCCTAAGGT CACTATCAGC

3 GTCAGAAACC ATTATTGCGC GTTCAAAAGT CGCCTAAGGT CACTATCAGC

p*NOS*

2101 2150

1 TAGCAAATAT TTCTTGTCAA AAATGCTCCA CTGACGTTCC ATAAATTCCC

2 TAGCAAATAT TTCTTGTCAA AAATGCTCCA CTGACGTTCC ATAAATTCCC

3 TAGCAAATAT TTCTTGTCAA AAATGCTCCA CTGACGTTCC ATAAATTCCC

p*NOS*

2151 2189 2200

1 CTCGGTATCC AATTAGAGTC TCATATTCAC TCTCAATCCA GATCCGGCCC

2 CTCGGTATCC AATTAGAGTC TCATATTCAC TCTCAATCCA GATCCGGCCC

3 CTCGGTATCC AATTAGAGTC TCATATTCAC TCTCAATCCA GATCCGGCCC

*nptII*

2201 2207 2250

1 ATGATCATGT GGATTGAACA AGATGGATTG CACGCAGGTT CTCCGGCCGC

2 ATGATCATGT GGATTGAACA AGATGGATTG CACGCAGGTT CTCCGGCCGC

3 ATGATCATGT GGATTGAACA AGATGGATTG CACGCAGGTT CTCCGGCCGC

*nptII*

2251 2300

1 TTGGGTGGAG AGGCTATTCG GCTATGACTG GGCACAACAG ACAATCGGCT

2 TTGGGTGGAG AGGCTATTCG GCTATGACTG GGCACAACAG ACAATCGGCT

3 TTGGGTGGAG AGGCTATTCG GCTATGACTG GGCACAACAG ACAATCGGCT

*nptII*

2301 2350

1 GCTCTGATGC CGCCGTGTTC CGGCTGTCAG CGCAGGGGCG CCCGGTTCTT

2 GCTCTGATGC CGCCGTGTTC CGGCTGTCAG CGCAGGGGCG CCCGGTTCTT

3 GCTCTGATGC CGCCGTGTTC CGGCTGTCAG CGCAGGGGCG CCCGGTTCTT

*nptII*

2351 2400

1 TTTGTCAAGA CCGACCTGTC CGGTGCCCTG AATGAACTGC AGGACGAGGC

2 TTTGTCAAGA CCGACCTGTC CGGTGCCCTG AATGAACTGC AGGACGAGGC

3 TTTGTCAAGA CCGACCTGTC CGGTGCCCTG AATGAACTGC AGGACGAGGC

2401 2450

*nptII*

1 AGCGCGGCTA TCGTGGCTGG CCACGACGGG CGTTCCTTGC GCAGCTGTGC

2 AGCGCGGCTA TCGTGGCTGG CCACGACGGG CGTTCCTTGC GCAGCTGTGC

3 AGCGCGGCTA TCGTGGCTGG CCACGACGGG CGTTCCTTGC GCAGCTGTGC

*nptII*

2451 2500

1 TCGACGTTGT CACTGAAGCG GGAAGGGACT GGCTGCTATT GGGCGAAGTG

2 TCGACGTTGT CACTGAAGCG GGAAGGGACT GGCTGCTATT GGGCGAAGTG

3 TCGACGTTGT CACTGAAGCG GGAAGGGACT GGCTGCTATT GGGCGAAGTG

*nptII*

2501 2550

1 CCGGGGCAGG ATCTCCTGTC ATCTCACCTT GCTCCTGCCG AGAAAGTATC

2 CCGGGGCAGG ATCTCCTGTC ATCTCACCTT GCTCCTGCCG AGAAAGTATC

3 CCGGGGCAGG ATCTCCTGTC ATCTCACCTT GCTCCTGCCG AGAAAGTATC

*nptII*

2551 2600

1 CATCATGGCT GATGCAATGC GGCGGCTGCA TACGCTTGAT CCGGCTACCT

2 CATCATGGCT GATGCAATGC GGCGGCTGCA TACGCTTGAT CCGGCTACCT

3 CATCATGGCT GATGCAATGC GGCGGCTGCA TACGCTTGAT CCGGCTACCT

*nptII*

2601 2650

1 GCCCATTCGA CCACCAAGCG AAACATCGCA TCGAGCGAGC ACGTACTCGG

2 GCCCATTCGA CCACCAAGCG AAACATCGCA TCGAGCGAGC ACGTACTCGG

3 GCCCATTCGA CCACCAAGCG AAACATCGCA TCGAGCGAGC ACGTACTCGG

*nptII*

2651 2700

1 ATGGAAGCCG GTCTTGTCGA TCAGGATGAT CTGGACGAAG AGCATCAGGG

2 ATGGAAGCCG GTCTTGTCGA TCAGGATGAT CTGGACGAAG AGCATCAGGG

3 ATGGAAGCCG GTCTTGTCGA TCAGGATGAT CTGGACGAAG AGCATCAGGG

*nptII*

2701 2750

1 GCTCGCGCCA GCCGAACTGT TCGCCAGGCT CAAGGCGCGC ATGCCCGACG

2 GCTCGCGCCA GCCGAACTGT TCGCCAGGCT CAAGGCGCGC ATGCCCGACG

3 GCTCGCGCCA GCCGAACTGT TCGCCAGGCT CAAGGCGCGC ATGCCCGACG

*nptII*

2751 2800

1 GCGAGGATCT CGTCGTGACC CATGGCGATG CCTGCTTGCC GAATATCATG

2 GCGAGGATCT CGTCGTGACC CATGGCGATG CCTGCTTGCC GAATATCATG

3 GCGAGGATCT CGTCGTGACC CATGGCGATG CCTGCTTGCC GAATATCATG

2801 2850

*nptII*

1 GTGGAAAATG GCCGCTTTTC TGGATTCATC GACTGTGGCC GGCTGGGTGT

2 GTGGAAAATG GCCGCTTTTC TGGATTCATC GACTGTGGCC GGCTGGGTGT

3 GTGGAAAATG GCCGCTTTTC TGGATTCATC GACTGTGGCC GGCTGGGTGT

*nptII*

2851 2900

1 GGCGGACCGC TATCAGGACA TAGCGTTGGC TACCCGTGAT ATTGCTGAAG

2 GGCGGACCGC TATCAGGACA TAGCGTTGGC TACCCGTGAT ATTGCTGAAG

3 GGCGGACCGC TATCAGGACA TAGCGTTGGC TACCCGTGAT ATTGCTGAAG

*nptII*

2901 2950

1 AGCTTGGCGG CGAATGGGCT GACCGCTTCC TCGTGCTTTA CGGTATCGCC

2 AGCTTGGCGG CGAATGGGCT GACCGCTTCC TCGTGCTTTA CGGTATCGCC

3 AGCTTGGCGG CGAATGGGCT GACCGCTTCC TCGTGCTTTA CGGTATCGCC

*nptII*

2951 3000

1 GCTCCCGATT CGCAGCGCAT CGCCTTCTAT CGCCTTCTTG ACGAGTTCTT

2 GCTCCCGATT CGCAGCGCAT CGCCTTCTAT CGCCTTCTTG ACGAGTTCTT

3 GCTCCCGATT CGCAGCGCAT CGCCTTCTAT CGCCTTCTTG ACGAGTTCTT

3001 3050

1 CTGAGCGGGA CTCTGGGGTT CGAAATGACC GACCAAGCGA CGCCCAACCT

2 CTGAGCGGGA CTCTGGGGTT CGAAATGACC GACCAAGCGA CGCCCAACCT

3 CTGAGCGGGA CTCTGGGGTT CGAAATGACC GACCAAGCGA CGCCCAACCT

3051 3100

1 GCCATCACGA GATTTCGATT CCACCGCCGC CTTCTATGAA AGGTTGGGCT

2 GCCATCACGA GATTTCGATT CCACCGCCGC CTTCTATGAA AGGTTGGGCT

3 GCCATCACGA GATTTCGATT CCACCGCCGC CTTCTATGAA AGGTTGGGCT

3101 3150

1 TCGGAATCGT TTTCCGGGAC GCCGGCTGGA TGATCCTCCA GCGCGGGGAT

2 TCGGAATCGT TTTCCGGGAC GCCGGCTGGA TGATCCTCCA GCGCGGGGAT

3 TCGGAATCGT TTTCCGGGAC GCCGGCTGGA TGATCCTCCA GCGCGGGGAT

partial t*OCS*

3151 3178 3200

1 CTCATGCTGG AGTTCTTCGC CCACCCCCTG CTTTAATGAG ATATGCGAGA

2 CTCATGCTGG AGTTCTTCGC CCACCCCCTG CTTTAATGAG ATATGCGAGA

3 CTCATGCTGG AGTTCTTCGC CCACCCCCTG CTTTAATGAG ATATGCGAGA

partial t*OCS*

3201 3250

1 CGCCTATGAT CGCATGATAT TTGCTTTCAA TTCTGTTGTG CACGTTGTAA

2 CGCCTATGAT CGCATGATAT TTGCTTTCAA TTCTGTTGTG CACGTTGTAA

3 CGCCTATGAT CGCATGATAT TTGCTTTCAA TTCTGTTGTG CACGTTGTAA

partial t*OCS*

3251 3304

1 AAAAACCTGA GCATGTGTAG CTCAGATCCT TACCGCCGGT TTCGGTTCAT TCTA

2 AAAAACCTGA GCATGTGTAG CTCAGATCCT TACCGCCGGT TTCGGTTC

3 AAAAACCTGA GCATGTGTAG CTCAGATCCT TACCGCCGGT TTCGGTTCAT TCTA

**Supplementary Figure S2: Pairwise alignment of the deduced amino acid sequences of *DFR* cDNA clones of *Petunia* × *hybrida* (NCBI X15537) and *Zea mays* (A_1_, NCBI CAA28734).**
The underlined amino acids represent the 26 amino acid stretch highlighted by Johnson et al. (2001) as putative substrate specificity determining region. The amino acids exchanged by site-directed mutagenesis in this work are highlighted by grey shades.

1 50

A1 MEGGAGASEK GTVLVTGASG FAGSWLVMKL LQAGYTVRAT VRDPANVGKT

PetuniaX15537 ..MPLHLRCS ATVCVTGAAG FIGSWLVMRL LERGYNVHAT VRDPENKKKV

51 100

A1 KPLMDLPGAT ERLSIWKADL AEEGSFHDAI RGCTGVFHVA TPMDFLSKDP

PetuniaX15537 KHLLELPKAD TNLTLLKADL TVEGSFDEAI QGCQGVFHVA TPMDFESKDP

101 150

A1 ENEVIKPTVE GMISIMRACK EAGTVRRIVF TSSAGTVNLE ERQRPVYDEE

PetuniaX15537 ENEVIKPTVR GMLSIIESCA KANTVKRLVF TSSAGTLDVQ EQQKLFYDQT

151 200

A1 SWTDVDFCRR VKMTGWMYFV SKTLAEKAAL AYAAEHGLDL VTIIPTLVVG

PetuniaX15537 SWSDLDFIYA KKMTGWMYFA SKILAEKAAM EEAKKKNIDF ISIIPPLVVG

201 250

A1 PFISASMPPS LITALALITG NAPHYSILKQ VQLIHLDDLC DAEIFLFENP

PetuniaX15537 PFITPTFPPS LITALSLITG NEAHYCIIKQ GQYVHLDDLC EAHIFLYEHP

251 300

A1 AAAGRYVCSS HDVTIHGLAA MLRDRYPEYD VPQRFPGIQD DLQPVRFSSK

PetuniaX15537 KADGRFICSS HHAIIYDVAK MVREKWPEYY VPTEFKGIDK DLPVVSFSSK

301 350

A1 KLQDLGFTFR YKTLEDMFDA AIRTCQEKGL IPLATAAGGD GFASVRAPGE

PetuniaX15537 KLTDMGFQFK Y.TLEDMYKG AIDTCRQKQL LPFSTRSAED NGHNREAIAI

351 376

A1 TEATIGA... .......... ......

PetuniaX15537 SAQNYASGKE NAPVANHTEM LSNVEV

**Supplementary Figure S3: RNA expression values of genes related to colour formation according to Bombarly et al. (2016) in *Petunia* × *hybrida* for cv. Electric Orange, cv. Salmon Ray, cv. Viva Orange, cv. Blackberry and cv. Corso Rot using the *Petunia axillaris* genome as reference.**
The green numbers represent the FPKM (Fragments Per Kilobase of transcript per Million mapped reads) values of the reads after normalization. A: full gene set; B: selection of structural genes ordered according to the phenylpropanoid/flavonoid pathway for better overview (next page).


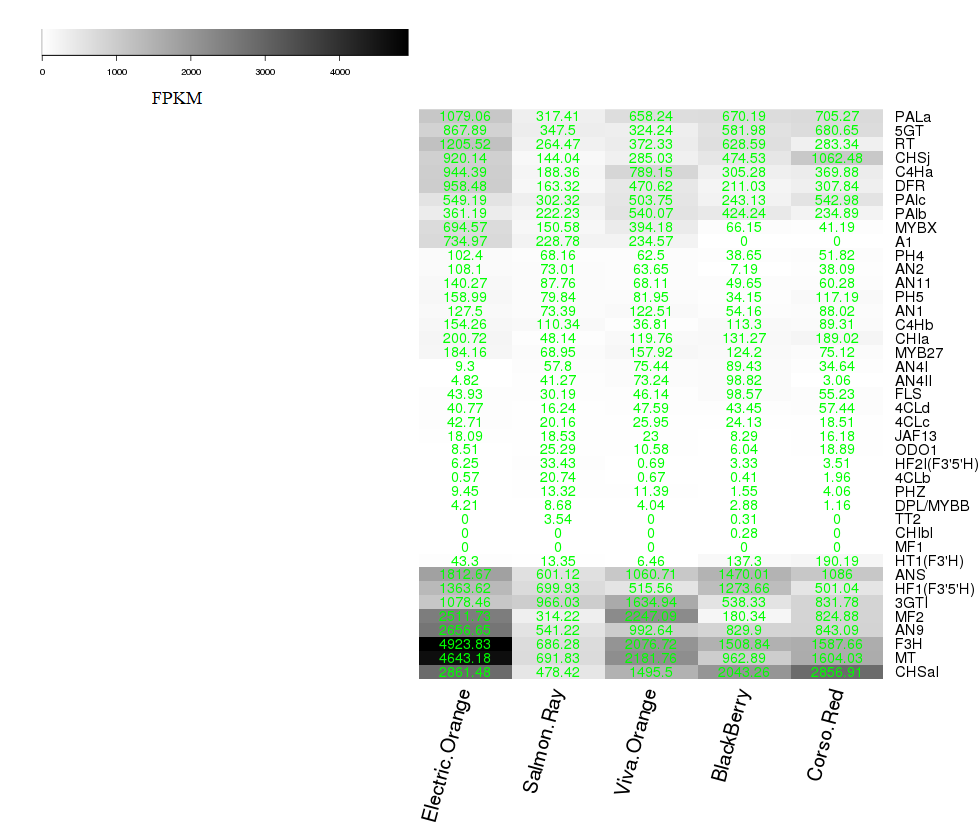

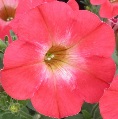

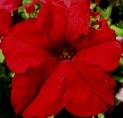

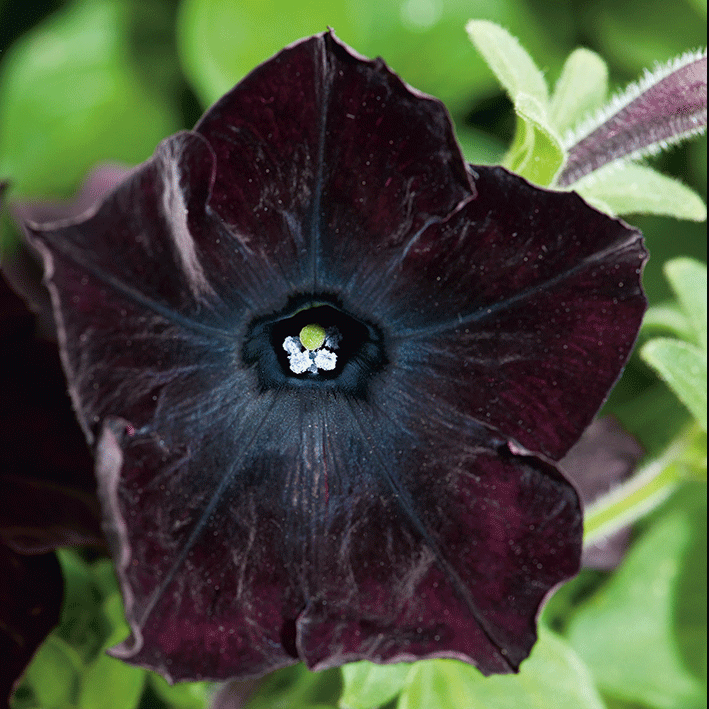

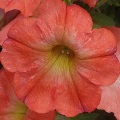

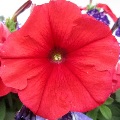


B

A

Genes encode: *3GT*: 3-glucosyltransferase; *4CL*: 4-coumaroyl:CoA-ligase; *5GT*: 5-glucosyltransferase; *A_1_*: dihydroflavonol 4-reductase of *Zea mays, AN1*: bHLH regulatory protein anthocyanin 1 ; *AN2*: MYB protein expressed in limb and tubes; AN4: MYB protein essential for pigmentation in anthers; AN9: Glutathione S-Transferase; AN11: WDR protein; *ANS*: anthocyanidin synthase; *C4H*: cinnamate-4-hydroxylase; *CHI*: chalcone isomerase; *CHS*: chalcone synthase; *DPL*: deep purple; regulatory gene with impact on pigmentation in vegetative tissues; *DFR*: dihydroflavonol 4-reductase; *F3H*: flavanone 3-hydroxylase; *FLS*: flavonol synthase; *HF2*: Flavonoid 3’5’-hydroxylase (F3’5’H); *HT1*: Flavonoid 3’-hydroxylase (F3’H); *JAF*: regulatory protein; *MF*: 3’5’-methyl transferase; *MT*: 3’-methyltransferase; *MYB*: transcription factor belonging to the MYB (myeloblastosis) family; *ODO*: scent regulator; *PH4*: vacuolar pH regulator; *PH5*: proton pumping activity; *PAL*: phenylalanine ammonium lyase; *PHZ*: purple haze, regulatory gene with impact on pigmentation in vegetative tissues; *RT*: rhamnosyltransferase; *TT2*: TRANSPARENT TESTA (MYB protein required for tannin accumulation);


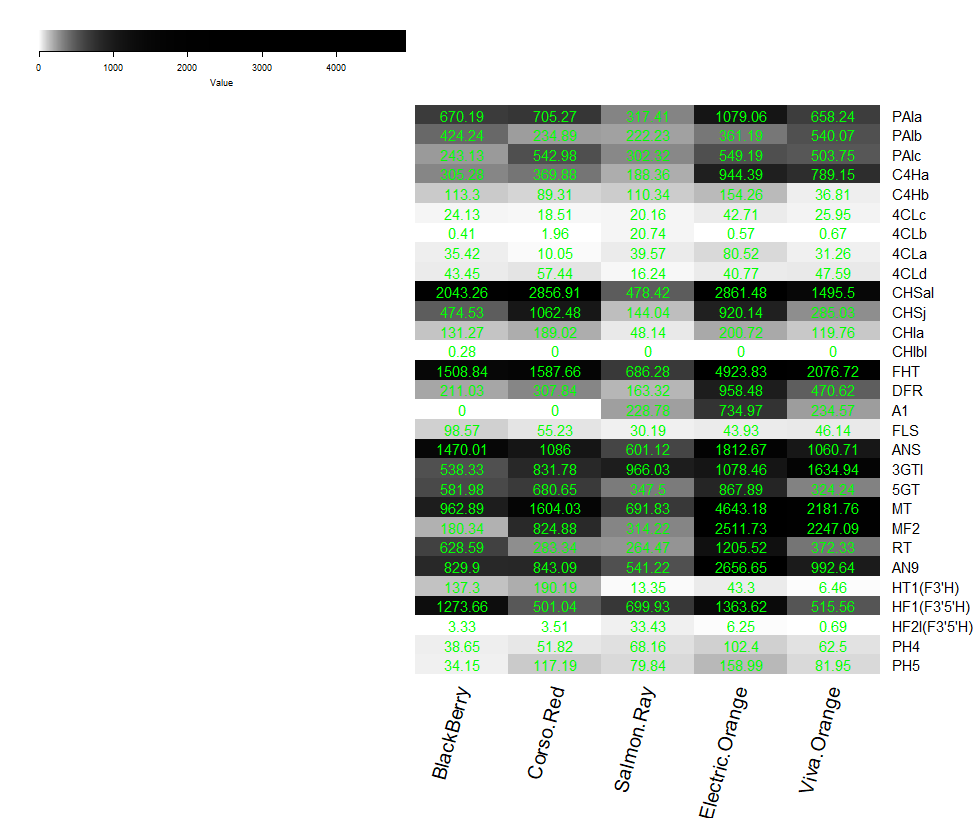


B


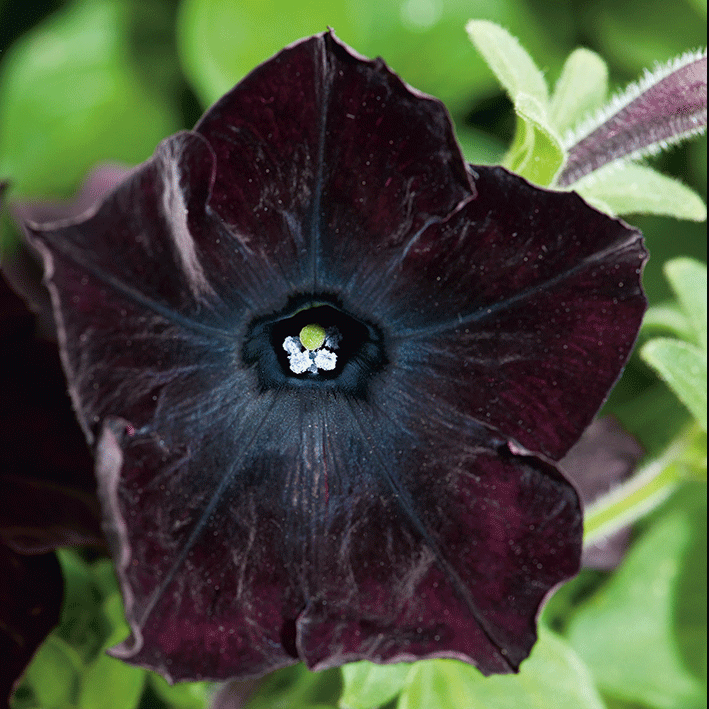

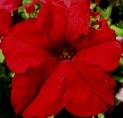

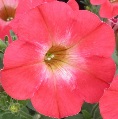

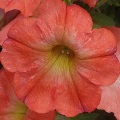

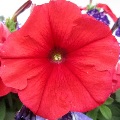


**Evaluation of the housekeeping gene for qPCR studies:**

For our results, qPCR data are particularly important to confirm indications of the transcriptome data and to investigate gene expression of selected flavonoid genes relative to each other in the three developmental stages of the three varieties. In this case, the influence of the reference gene is almost negligible as it is eliminated in e.g. *A_1_:PhDFR* gene expression ratios. We nevertheless made strong efforts to choose a reliable reference gene and screened primers for reference genes used in literature for *Petunia* sp. (Mallona, 2010). The majority showed insufficient specificity based on the melt curves we obtained and were therefore sorted out. We evaluated two candidates of reference genes, *actin* (Suppl. Table S1) and *SAND* (Mallona, 2010) with respect to expression stability and quality of signals. The two reference genes showed somewhat differing absolute values in the expression during flower development but both confirmed the very low gene expression of *F3'H* and *FLS* in comparison to *PhDFR* in the tested samples*.* Based on this results, we used *actin*, as reference gene, because it showed best melting curves and lower C_t_-values than *SAND*.
